# Cross-disciplinary “General Learning Objectives” for postgraduate medical training: Prioritisation of five competency domains using a Delphi process

**DOI:** 10.3205/zma001875

**Published:** 2026-09-15

**Authors:** Elke Bayha, Hermann Armstad, Giatgen Spinas, Monika Brodmann Maeder, Eva K. Hennel

**Affiliations:** 1 SIWF Schweizerisches Institut für ärztliche Weiter- und Fortbildung, Bern, Switzerland; 2 Universität Bern, Institut für Medizinische Lehre (IML), Bern, Switzerland; 3 Universität Bern, Institut für Medizinische Lehre (IML), Master of Medical Education Program (MME), Bern, Switzerland; 4 amstad-kor, Basel, Switzerland

**Keywords:** competency-based education, intrinsic CanMEDS roles, postgraduate medical education, learning objectives, Delphi process

## Abstract

**Objective:**

The Swiss postgraduate education regulations stipulate that teaching be guided by cross-disciplinary “General Learning Objectives” (GLOs) based on the CanMEDS roles. Notwithstanding these regulations, teaching activities and performance criteria for GLOs are scarcely evident in postgraduate training programmes. The primary aim of this study was to determine the reasons why too little emphasis is placed on GLOs in postgraduate training programmes. A secondary aim was to define a reduced set of needs-based cross-disciplinary competencies that trainees should develop during their postgraduate medical training.

**Methodology:**

A Delphi study was conducted to survey postgraduate trainees and heads of training centres throughout Switzerland. In two rounds of surveys, data were collected on (1) why too little emphasis is placed on GLOs in postgraduate training and (2) which GLOs are considered relevant. Based on these results, the GLOs were then consolidated into competency domains.

**Results:**

The main reason for the underemphasis on GLOs was the high workload of postgraduate trainees. Based on the data from both survey rounds, the following five competency domains were formulated and prioritised by the research team: “patient safety”, “communication in physician-patient relationships”, “mental health of physicians”, “collaboration in interprofessional teams” and “clinical ethics”.

**Conclusions:**

The results of the Delphi study confirm the need for action and provide important insights into how sub-aspects of GLOs are weighted by clinicians. Barriers to implementation of GLOs include the high workload imposed on postgraduate trainees and the lack of awareness about GLOs. The final concept comprised five cross-disciplinary competency domains that provide an application-oriented framework to underpin postgraduate medical training.

## Zusammenfassung

**Zielsetzung:**

Die ärztliche Weiterbildungsordnung (WBO) der Schweiz verlangt die Vermittlung von überfachlichen „Allgemeinen Lernzielen“ (ALZ), die auf den CanMEDS-Rollen basieren. Unterricht und Leistungskriterien der ALZ sind in den Weiterbildungsprogrammen jedoch kaum sichtbar. Ziel dieser Studie ist es, die Gründe für die geringe Beachtung der ALZ zu ermitteln und eine reduzierte Anzahl bedarfsgerechter fachübergreifender Kompetenzen für die Vermittlung in der Weiterbildung zu formulieren.

**Methodik:**

In einem Delphi-Verfahren wurden Ärzt:innen in Weiterbildung und Leiter:innen von Weiterbildungsstätten schweizweit befragt. In zwei Umfragerunden wurde erhoben, (1) warum die ALZ in der Weiterbildung eine geringe Beachtung finden und (2) welche ALZ als relevant beurteilt werden. Unter Berücksichtigung der Ergebnisse wurden die ALZ anschliessend zu Kompetenzbereichen verdichtet.

**Ergebnisse:**

Als wichtigster Grund für die geringe Beachtung der ALZ wird die hohe Arbeitsbelastung der Ärzt:innen in Weiterbildung genannt. Basierend auf den Daten beider Befragungen wurden im Forscherteam folgende fünf Kompetenzbereiche formuliert und priorisiert: „Patientensicherheit“, „Kommunikation in der Arzt-Patientenbeziehung“, „mentale Gesundheit von Ärzt:innen“, „Zusammenarbeit im interprofessionellen Team“ und „klinische Ethik“.

**Schlussfolgerung:**

Die Ergebnisse des Delphi-Verfahrens bestätigen den Handlungsbedarf und liefern wichtige Erkenntnisse darüber, wie Teilaspekte der ALZ von Kliniker:innen gewichtet werden. Barrieren in der Umsetzung, sind die hohe Arbeitsbelastung von Ärzt:innen in Weiterbildung und die Unbekanntheit der ALZ. Das finale Konzept umfasst fünf fachübergreifende Kompetenzbereiche, die ein anwendungsorientiertes Rahmenkonzept für die ärztliche Weiterbildung darstellen.

## 1. Introduction

The General Learning Objectives (GLOs) set forth in the Swiss postgraduate education regulations [1] define cross-disciplinary standards that are compulsory for the postgraduate training programmes of all medical specialities. A catalogue of learning objectives describes these standards in seven physician roles [2] based on the CanMEDS model [3]. The Swiss model stipulates that the medical speciality societies are responsible for all learning content that is purely specific to that medical speciality. In this context, such speciality-specific learning content is primarily reflected in the CanMEDS role of “medical expert”. The cross-disciplinary GLOs are primarily situated in the six other roles referred in the literature as intrinsic CanMEDS roles.

The Swiss postgraduate education regulations require that the medical speciality societies and postgraduate training centres emphasise GLOs alongside the learning content specific to each discipline. However, attendance at teaching activities specifically on GLOs is optional and proper curricular integration of GLOs is not monitored [4]. In retrospect, physicians remarked that the way many of the GLOs were covered during their postgraduate education was unsatisfactory or barely satisfactory [5]. Furthermore, the performance of postgraduate trainees is not documented in sufficient detail on the postgraduate education certificate (cf. attachment 1 ). This certificate contains 22 competencies required by the Swiss specialist training regulations that reflect the GLOs from the catalogue of learning objectives as thematic areas (see attachment 2 ) and competency is rated on a four-point scale (ranging from 1=strongly agree to 4=strongly disagree).

Not only in Switzerland, the implementation and assessment of intrinsic CanMEDS roles pose a challenge in competency-based postgraduate training. Also on an international scale, speciality-specific competencies compete with intrinsic CanMEDS competencies [6], [7], [8].

In the past, there had already been criticism about how GLOs were implemented in postgraduate education curricula [5], [9], [10]; initiatives have been launched to raise awareness of relevant cross-disciplinary competencies and how they can be made more explicit in postgraduate education [4], [11]]. Besides the comprehensive version of the catalogue of learning objectives for postgraduate education programmes and the 22 competencies required by the Swiss specialist training regulations, the Swiss Institute of Medical Education (SIME) has issued a binding recommendation requiring that eight partially new topics like “health economics and management” and “leadership” be integrated into postgraduate education curricula [4]. This has caused the current representation of the GLOs in the SIME requirements to appear overloaded, fragmented and redundant. We suspected this was one of the reasons why their implementation has been less successful. That situation spurred the launch of a national project in Switzerland to promote cross-disciplinary competencies and improve their implementation.

This study examines how postgraduate trainees and postgraduate training centres perceive the implementation and substantively emphasise existing GLOs. With the aim to identify a feasible number of cross-disciplinary competencies tailored to the needs of postgraduate medical education, our research poses the following questions:

1. Why is too little emphasis placed on GLOs in postgraduate medical training?
2. Which of the existing GLOs and which new competencies should be integrated into and assessed in postgraduate medical training curricula in the future?

## 2. Methods

The different ways that SIME presents the GLOs form the starting point for the two-part methodological approach to prioritisation of GLOs (see figure 1 (Fig. 1)).

### 2.1. Delphi process

#### 2.1.1. Reporting and objectives

Our methodology involved two survey rounds (see figure 2 (Fig. 2)) covering the five core features of typical Delphi processes [12]. These core features are anonymity, at least two Delphi rounds, a standardised questionnaire, statistical analysis based on descriptive data and feedback on the 2^nd^ round’s results. We used the DELPHISTAR recommendations for standardised reporting (see attachment 3 ) [13]. By reaching a consensus on the relevance of existing GLOs and potentially new topics, we aim to improve the evidence on and raise awareness of necessary, cross-disciplinary educational standards for postgraduate trainees.

#### 2.1.2. Questionnaire

The questionnaire for the 1st round was developed on the basis of previous survey data, a random-sample analysis of performance assessments and interviews with representatives of various stakeholder groups. Next, the questionnaire was revised by the research team (see attachment 4 ). For practical reasons (consistency, scope, degree of recognition), the questionnaire was based on the 22 competencies required by the Swiss specialist training regulations listed on the postgraduate education certificate. In the context of this study, these competencies are considered synonymous with the GLOs. The questionnaire included questions on socio-demographic data and assessment of the status quo of GLOs in postgraduate education (research question 1: questions 6-8), to assess the existing GLOs and other possible topics (research question 2: questions 9-52 on the existing GLOs; questions 53-68 on new topics). Closed-ended question formats were combined with the option to enter comments or new topics in free-text fields. The answers to questions 6-8 were intended for information purposes only. That was why there was no consensus criterion for this part. For questions 9-62, consensus was reached if at least 60% of respondents rated the item as “very relevant”. New topics were included if they were mentioned more than 20 times in total.

For the 2^nd^ round, the questionnaire was revised and the number of questions reduced (see attachment 5 ). The reduction was intended to maintain the respondents’ motivation to participate. No further questions were asked about research question 1. The questions on the respondents’ socio-demographic situation were retained and supplemented by a question regarding their participation in the 1^st^ round. The assessment of the topics that should be covered in postgraduate medical training was carried out using a matrix in which sixteen topics were plotted on the vertical axis against eight priorities in descending order on the horizontal axis (research question 2: question 7). No consensus criteria were defined in advance; instead, it was planned to make a post-hoc decision after review of the results in order to keep the options open regarding practical feasibility. No 3^rd^ round was administered due to participant response rates.

#### 2.1.3. Implementation

Contrary to the conventional Delphi methods, this study comprised an exhaustive expert sampling. The aim of this modification was to ensure that data collection covered the broadest possible base. As primary stakeholders, all Swiss postgraduate trainees and heads of postgraduate training centres are regarded as experts. At the time of the survey, the questionnaire was sent to 14.130 postgraduate trainees and 3.901 heads of postgraduate training centres. Prior to the first round, articles were published in the journal *Schweizerische Ärztezeitung* and the vsao Journal of the Association of Swiss Junior and Senior Residents announcing the surveys along with explanations of the reasons behind them [14], [15]. The e-mail text for the second survey round included a link to the results of the first survey round, which were published on the SIME website [16]. The online questionnaires were posted on the survey platform of SurveyMonkey Europe UC [https://de.surveymonkey.com/] and the link to the survey sent by e-mail. E-mail and questionnaire were provided in both German and French. Participation in the Delphi study was voluntary and anonymous. The first round of the online survey was open from 22 June to 17 July 2023; the 2nd round ran from 19 September to 16 October 2023.

#### 2.1.4. Data analysis

Only fully completed questionnaires were included in the analysis. The statistical analysis was carried out using Excel. To analyse the results of the 2^nd^ round, the priorities assigned were converted into scores (1^st^ priority=8 points, 2^nd^ priority=7 points etc.) and totalled. The means were calculated by dividing the total points for each item by the number of survey respondents. One member of the research team synthesised the qualitative data by topic (free-text responses on other topics from questionnaire 1 and the field for other comments from both rounds) and counted the number of topics. No coding or categorisation was required.

### 2.2. Ethical approval

The Cantonal Ethics Committee of Bern ruled that this project was not subject to approval under the Swiss Federal Human Research Act (Req-2023-00602).

### 2.3. Conceptualisation within the research team

The results of the Delphi method were organized by theme and revised by the research team (the entire team, consisting of five people) in several meetings. During its discussions, the research team became aware of the formal contradictions mentioned in the results. The conceptualisation process was not planned in advance, nor did it follow a fixed structure, but took into account the priority of the topics and any potential overlap. The aim was to narrow down the topics to such an extent that made their implementation realistic in a limited number of smaller, content-focused projects. To this end, the team discussed which aspects could be grouped together thematically. In addition, insights gained from several workshops on the topic of “general learning objectives” were incorporated. These workshops took place, for example, at the annual medical education symposium “med-ed-symposium” organised by the SIME, where selected medical education experts chose the topics they considered most important in their clinical routine. Overall, the team decided to deviate from the Delphi result and their iterative discussion rounds resulted in five competency domains.

### 2.4. Research team and authors’ contributions to study

Elke Bayha (EB) is project lead for “GLOs in postgraduate medical education” and “a quality label for medical education” at the SIME with experience in competency-based curriculum development and the Swiss Medical Federal Licensing Exam; she was primarily responsible for literature research, data interpretation and drafting the discussion and the manuscript. Hermann Amstad (HA) is a former secretary-general of the Swiss Academy of Medical Sciences (SAMW) and a health policy adviser; he developed the questionnaire and managed the data collection process. Giatgen Spinas (GS) was director of a postgraduate training centre being part of a university hospital and is vice-president of SIME; he supported the data interpretation process. Monika Brodmann Maeder (MBM) was president of the SIME and managed the Delphi process. Eva Hennel (EH) is head of research at SIME, managed the data interpretation process and helped draft the manuscript.

## 3. Results

### 3.1. Participant characteristics

The response rate of fully completed questionnaires was 11% (n=2,028) in the first round and 8% (n=1,391) in the 2^nd^ round. The response rate, including partially completed questionnaires and the resulting exclusions, is presented in figure 2 (Fig. 2). The socio-demographic characteristics of the participants in both rounds are presented in table 1 (Tab. 1).

### 3.2. Reasons why too little emphasis is placed on GLOs in postgraduate medical training (research question 1)

The majority of respondents agreed that too little emphasis is placed on GLOs in postgraduate medical training (56.4% “yes”, 23.5% “no”, 20.1% “do not know”). They attributed this to the heavy workload imposed on postgraduate trainees (73.8%) and to a lack of recognition of the GLOs (47.3%). It is also unclear how the achievement of the GLOs should be verified (44.4%). By contrast, only 2.6% of respondents believed that the GLOs were unnecessary. The results of the responses to question 7 are presented in table 2 (Tab. 2).

### 3.3. Prioritisation and reduction of the GLOs for postgraduate education (research question 2)

In the first round (questions 9-62), twelve of the GLOs listed were rated by at least 60% of respondents as “very relevant” and therefore planned for inclusion in the 2^nd^ round. Four new topics (“work-life balance”, “smarter medicine”, “migration/transcultural competency“, “teaching/leadership”) were proposed by more than 20 participants and likewise included. The detailed results of the 1^st^ round were published on the SIME website [16]. The 2^nd^ round of the Delphi process was intended to validate the results of the 1^st^ round and further reduce the number of competencies. Table 3 (Tab. 3) presents the ranking by mean score achieved across the overall group, by language, gender, professional situation and participation in the 1^st^ round. There are no noteworthy differences between the responses across the various subgroups.

When reviewing the results of the Delphi surveys (see 2.3. Conceptualisation within the research team), the research team became aware of the following formal contradictions in the GLOs surveyed:

- The GLOs are formulated neither as learning objectives nor as competencies but represent thematic aspects of patient care.
- The GLOs show fundamental, substantive overlaps in terms of content, both among themselves and speciality-specific competencies defined by the medical speciality societies in their postgraduate education programmes.
- The levels of granularity of the terms used varies. Some terms encompass broad topics, such as “patient safety”, whilst others only describe a partial aspect like “recognizing one’s own limits”.

As a result of the conceptualisation process within the research team, these issues were adjusted as follows: The research team perceived the GLOs as competency domains that form a guideline for the identification of competencies and provide structure to the content. To resolve the issues concerning overlaps and levels of granularity, the GLOs from the 2^nd^ round were grouped into five competency domains:

1. Patient safety
2. Communication in physician-patient relationships
3. Collaboration in interprofessional teams
4. Mental health of physicians
5. Clinical ethics

These should be pursued for implementation in postgraduate medical training. The reasoning that led to the five competency domains is summarised in table 4 (Tab. 4). In the text, we confined ourselves to explaining the reasons that led us to consider competency domains 4 and 5 contrary to the survey results.

The GLO “physicians health” – which specifically refers to their mental health – achieved an above-average score in the 1^st^ survey and was carried over to the 2nd round as “Work-Life Balance.” The research team deliberated about how psychosocial stress factors like inadequate “Work-life balance” and “Self-organisation/structuredness” are closely linked to impaired health. Ultimately, however, they specify only certain aspects of mental health, which could also be influenced by other factors. It was hypothesised that the scores of “work-life balance” in the 2^nd^ round were lower because respondents associated other factors with physicians’ mental health and gave greater weight to them. These deliberations led to the inclusion of the competency domain “mental health of physicians”. The GLOs “work-life balance” and “self-organisation/structuredness” are now incorporated therein”.

The competency domain “clinical ethics” was regarded as a central aspect of the physician-patient relationship. The discussion also considered results from SAMW studies [17], [18] showing that the necessary structures and clinical-ethical expertise are not adequately developed at the institutions. For that reason and even though the “ethics” GLO received a low rating in the 1^st^ round, it was nonetheless included as the competency domain “clinical ethics” for postgraduate medical training.

## 4. Discussion

### 4.1. Contextual challenges in the implementation of cross-disciplinary competencies (research question 1) and recommended courses of action 

Elucidation of the first research question produced evidence on the contextual situation at postgraduate training centres in terms of attitudes towards existing GLOs and potential barriers to implementation. These findings underscore the need for action and help in devising interventions that elevate the status of GLOs. Our impression that the existing GLOs are not adequately emphasised in postgraduate education curricula has been confirmed. At the same time, the need for cross-disciplinary educational standards for postgraduate trainees has been highlighted. Given the complex contextual interactions that often impede implementation processes, particular attention should be paid to the clinical learning environment [19].

The high workload imposed on postgraduate trainees stands at the top of the list of reasons for the existing situation. Therefore, the implementation process should utilise synergies and be flanked by measures to relieve the burden on postgraduate trainees. Potential synergies comprise the integration of competencies into existing educational activities and curricula or the combination of mutually complementary content. Measures at the postgraduate training centres include ensuring that dedicated time is set aside for structured learning activities, that specific tasks are supervised and positive role models are prioritised as a means for trainees to acquire behaviours and competencies.

We regard the fact that the stakeholders in postgraduate education are largely unfamiliar with the GLOs and that the means by which curricular integration and evaluation of GLOs should take place remain unclear. This is further evidence that the complex clinical working environment – with its multilevel demands – does not prioritise the existing GLOs. This trend is likely reinforced by the many redundant and often incoherent descriptions of GLOs. A clearly formulated description of learning outcomes that is equally understandable to both faculty and learners is a prerequisite for implementation of cross-disciplinary competencies. It was therefore necessary to reduce the number of GLOs and present the overall concept in a more straightforward manner. Furthermore, successful practical implementation is subject to the availability of specific opportunities for learning, practicing, assessment and obtaining feedback. Although the publication titled “principles and recommendations for teaching GLOs in postgraduate medical education” from 2016 [4] had addressed these aspects, no binding regulations or specific proposals for GLO implementation have been established to date. To ensure that the current efforts to strengthen the implementation of cross-disciplinary competencies does not suffer the same fate, the medical speciality societies and postgraduate training centres must not be left to tackle this challenge on their own. There is a need for educational activities that allow internal implementation with low thresholds or can be supplemented by curricular activities that are available externally.

### 4.2. Prioritisation of cross-disciplinary competencies for postgraduate education (research question 2)

The Delphi study delivered valuable insights into which thematic priorities were considered relevant. In the 2^nd^ survey round, there were sixteen topics to choose from, eight of which were to be adopted. Upon reviewing the results within the research group, however, the following deviation from this procedure was made. Instead of the eight highest-priority topics, it was decided to consolidate almost all topics into competency domains (see table 4 (Tab. 4)). Exceptions involved those topics categorised as discipline-specific GLOs and the topic “teaching/leadership”. With a mean of only 1.3, the latter was not rated particularly highly. Furthermore, it was not considered by the research team to be a competency that should primarily be required of postgraduate trainees. In Switzerland, the competency domains labelled as discipline-specific in table 4 (Tab. 4) (“diagnostics and therapy”, “pharmacotherapy”, “smarter medicine”) are the responsibility of the medical speciality societies. It is therefore necessary to distinguish between discipline-specific and cross-disciplinary competencies in order to avoid redundancies or even contradictions between the requirements of SIME and those issued by the medical speciality societies.

The final framework, consisting of five cross-disciplinary competency domains, incorporates the respondents’ ratings. Four of the competency domains are derived from former existing GLOs, with “clinical ethics“ being retained based on the results of recent SAMW studies [17], [18] and contrary to the respondents’ weighting. Out of the newly proposed topics, only “mental health of physicians” was included in the new competency requirements. Over the further course of the national project, key competencies should be defined for each competency domain.

### 4.3. The CanMEDS model in the workplace-based clinical learning environment

In clinical routine, the CanMEDS concept is just one of myriad impact factors and hardly dictates the interaction between postgraduate trainees and their supervisors [6]. Intrinsic competencies are underrepresented in curricula, learning objectives and assessment data for postgraduate trainees [8], [20], [21]. In the workplace-based clinical learning environment, intrinsic roles are mainly conveyed through observation of role models and often remain implicit [21]. Although physicians consider these roles to be relevant, their description is not always perceived as clear or applicable or may not be associated with observable behaviour [7], [22]. Moreover, it was considered possible that postgraduate training staff finds it difficult to fully grasp the underlying constructs [20]. In practice, it seems rather artificial to divide the complex medical profession into seven roles because these roles overlap and complement each other [23]. On the other hand, competency frameworks can help reduce the cognitive load in clinical teaching by providing a structure for observations [6]. Here, we see possible explanations for the problems we encountered in the past regarding poor implementation of the GLOs for postgraduate education. Hence, it appears imperative that the CanMEDS model be integrated into postgraduate education in a way that is relevant to real-world applications. On the one hand, our competency domains are derived from the CanMEDS model and, on the other hand, reflect the expectations placed on postgraduate trainees from a practice-orientated perspective. We view this approach as a dynamic model that should be reviewed at regular intervals and adapted to evolving needs. As such, this study highlights one way in which the intrinsic CanMEDS roles can be adapted to clinical practice in order to foster their implementation. Our study thus makes a contribution that could also be applied to postgraduate education in other systems.

### 4.4. Learning objectives versus competencies

At the time the catalogue of learning objectives for postgraduate medical education was developed, the use of the term “general learning objectives” to describe cross-disciplinary competencies was a logical choice. The authors were guided by the existing “Swiss Catalogue of Learning Objectives for Undergraduate Medical Training” [24], a manual which uses the English expression “general objectives” to describe the CanMEDS roles. In the meantime, the understanding of “learning objective” versus “competency” has become even more nuanced. A learning objective defines the desired outcome of a teaching unit, whereas the concept of competency focuses on the learners. Therefore, the use of the term “learning objective” is not consistent with the principle of competency orientation. “General” leaves room for ambiguity regarding the nature of these competencies and does not specify that these are medical competencies that should apply equally to all specialties. That is why we recommend that the term “GLO” be replaced with a more appropriate descriptor as part of the reform process.

### 4.5. Strengths and limitations of this study

The Delphi method is a suitable way to gather expert opinions to inform the planning of interventions [25]. The use of the DELPHISTAR reporting guideline to illustrate the Delphi process gives transparency to our methodology. Our exhaustive expert sampling is a modification of conventional Delphi methods designed to provide a broader basis for supporting the results. Consequently, the group of experts was not identical in the 1^st^ and 2^nd^ Delphi rounds. The additional question in the 2^nd^ round questionnaire regarding participation in the 1^st^ round, shows the impact of this modification. The distribution of the respondents’ socio-demographic characteristics is comparable in both survey rounds. Moreover, the results of the first round were made accessible to the participants in the 2^nd^ round. After weighing all the factors, we decided on this process, partly with a view to increasing the stakeholders’ acceptance of the upcoming intervention. The survey of the two professional groups – postgraduate trainees and heads of postgraduate training centres – makes the results particularly valuable, as it takes into account the perspectives of both learners and supervisors alike. 

When developing the questionnaire, the research team decided to retain the GLO concept specified in the SIME certificate and required by Swiss specialist training regulations, instead of revising the GLOs thematically prior to the survey. The fact that conceptualisation within the research team took place at a later stage was one limitation of the consensus-building process but was necessary in order to ensure the project’s feasibility. Care was taken to ensure that the respondents’ preferences were not falsified. Any marked deviation from expert opinion was clearly indicated.

In this study, we have placed the GLOs for postgraduate medical training in alignment with the CanMEDS framework. Although the GLOs are organised by role, we are aware that the translation into German and the influence of other competency frameworks during their development may have meant that some contents of the Swiss document may deviate from the original. At this juncture, we would still like to point to our belief that the five competency domains continue to have a clear link to the CanMEDS model. Due to this complexity, most teachers and learners will not immediately grasp that connection. Nevertheless, this reference should be made clear to those involved in planning educational programs. To counteract even more fragmentation, we propose providing a brief holistic definition of the roles for postgraduate training to offer a comprehensive overview and referring readers to the original source for further details.

## 5. Conclusion

This study examined two research questions as part of a national project on cross-disciplinary competencies. The first research question on the situation at postgraduate training centres significantly contributed to an understanding of the status quo. Namely, that too little emphasis is placed on cross-disciplinary competencies in postgraduate education due to the high workload imposed on postgraduate trainees and because the degree of recognition for these competencies is low. Our example from Swiss postgraduate education raises awareness of the challenges involved in teaching values and behaviors in a workplace-based learning environment. The second research question led to the five cross-disciplinary competency domains “patient safety“, “communication in physician-patient relationships”, “collaboration in interprofessional teams”, “mental health of physicians” and “clinical ethics”. Health education professionals with specific expertise in one of these competency domains will now be tasked with requests to propose postgraduate education competencies and possible implementation formats. Subsequently, the intent is to follow-up with the development of a concept for teaching and performance evaluation of these competencies in close cooperation with representatives of the medical speciality societies and postgraduate training centres.

## Authors’ ORCIDs

- Elke Bayha: [0009-0003-6509-3949]
- Giatgen Spinas: [0009-0007-6142-2564]
- Monika Brodmann Maeder: [0000-0001-5608-7887]
- Eva K. Hennel: [0000-0002-7625-5785]

## Competing interests

The authors declare that they have no competing interests. 

## Supplementary Material

Certificate (Translation of SIWF-Zeugnis: WBO-Kompetenzen)

WBO-Kompetenzen und ihre Herleitung aus den Allgemeinen Lernzielen (only in German)

Fragebogen Delphistar (only in German)

Standardised questionnaire: General Learning Objectives – 1st round Delphi survey of postgraduate trainees and heads of postgraduate training centres

Standardised questionnaire: General Learning Objectives – 2nd round Delphi survey of postgraduate trainees and heads of postgraduate training centres

## Figures and Tables

**Table 1:**
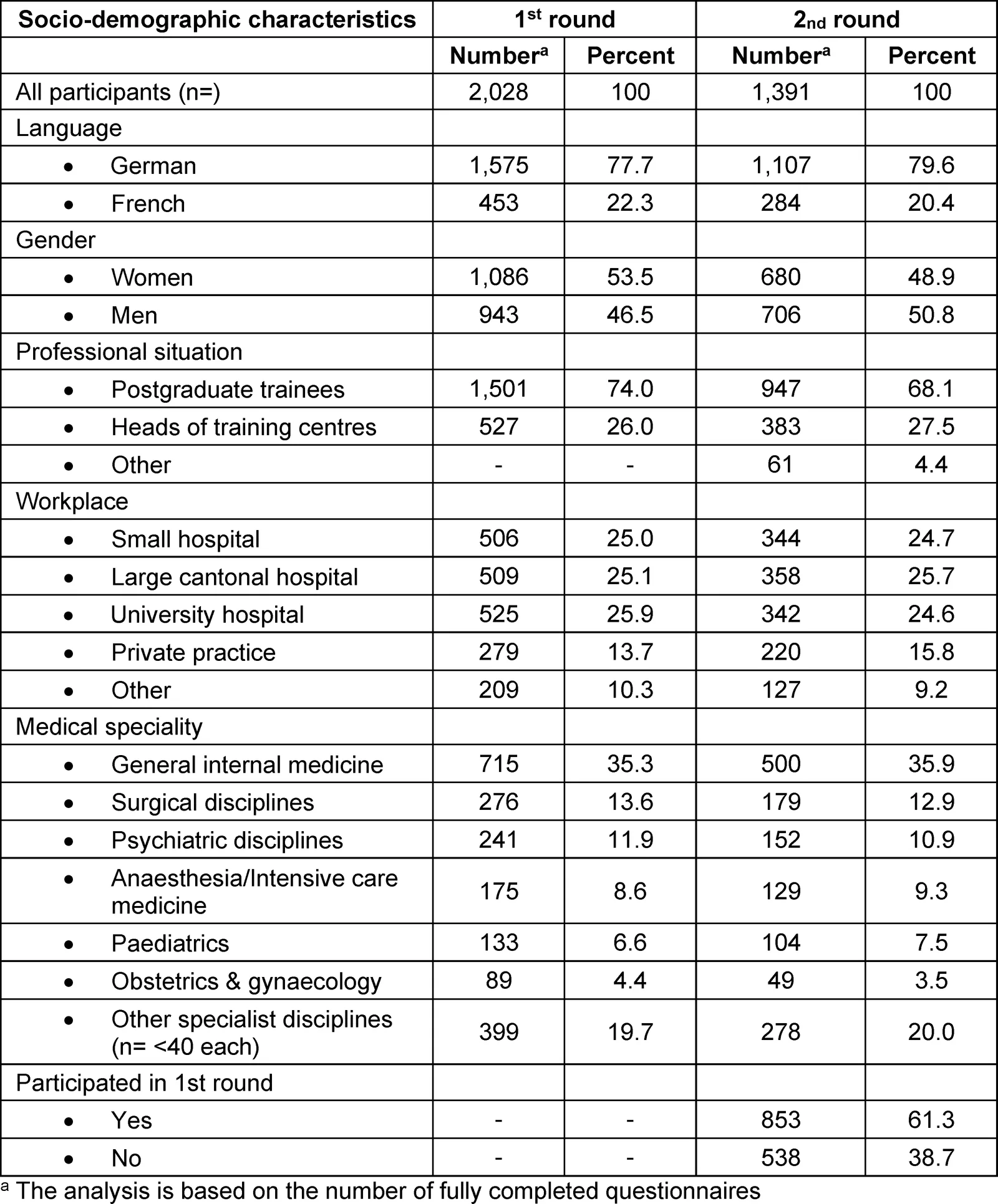
Socio-demographic characteristics of survey participants

**Table 2:**
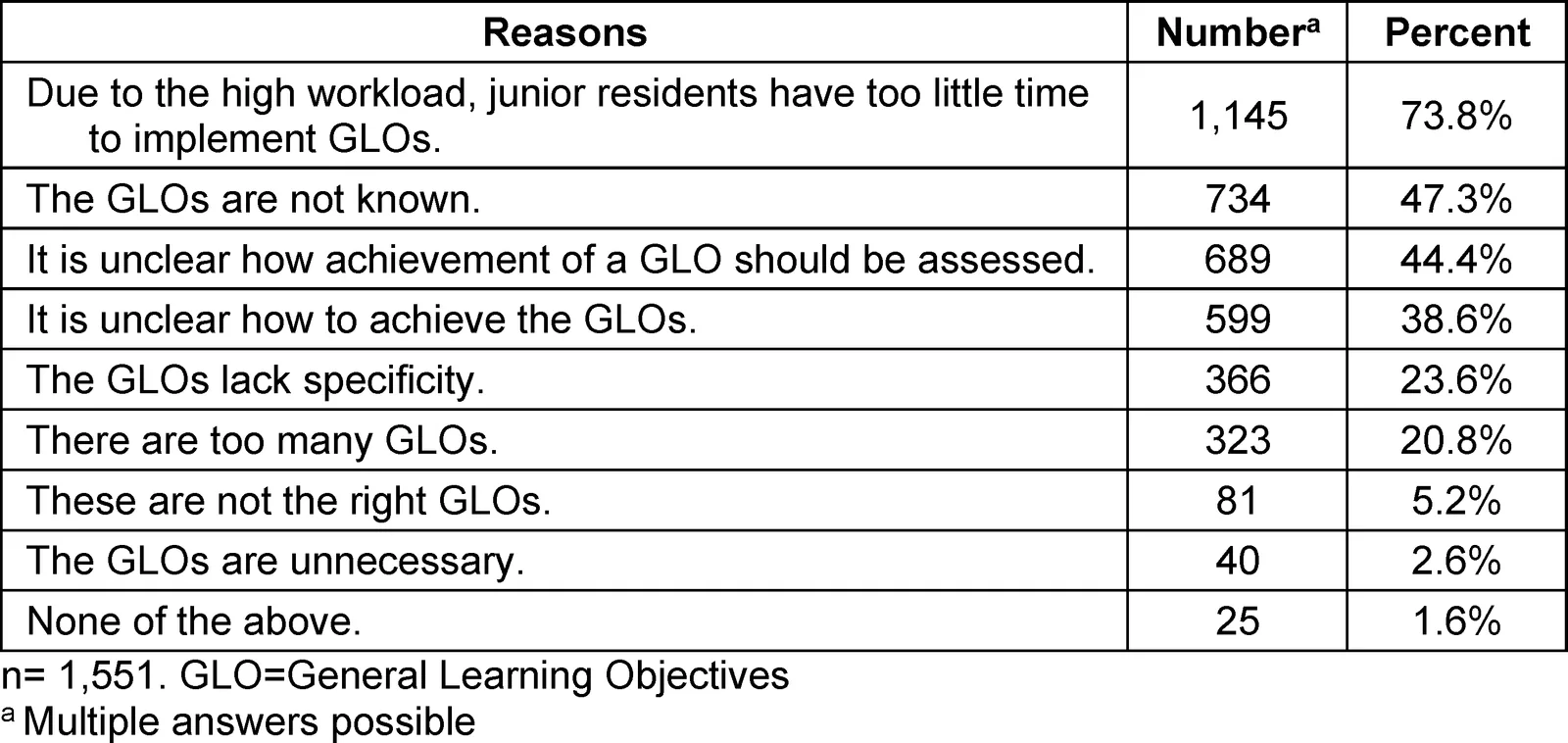
Reasons for the lack of emphasis on general learning objectives in descending order. Only those participants were questioned who answered “yes” or “do not know” to the question of whether there is too little emphasis on GLOs or not.

**Table 3:**
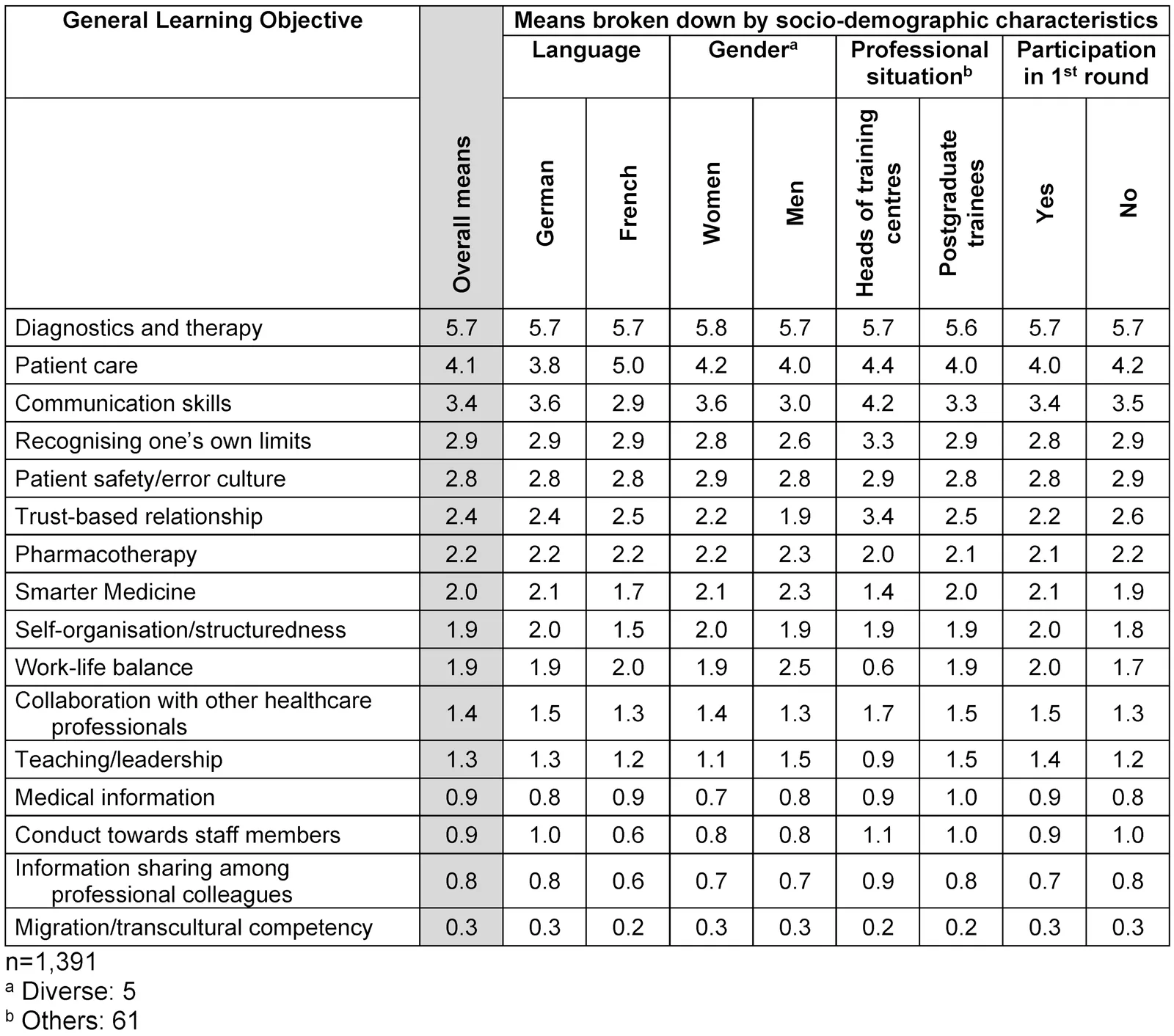
Ranking by means of the assessment of the GLOs by all survey respondents (sorted in descending order) and broken down by socio-demographic characteristics. The set priorities were converted to scores (1^st^ priority=8 points; 2^nd^ priority=7 points etc.) and totalled. To calculate the means, the total points assigned to one GLO were divided by the number of survey respondents.

**Table 4:**
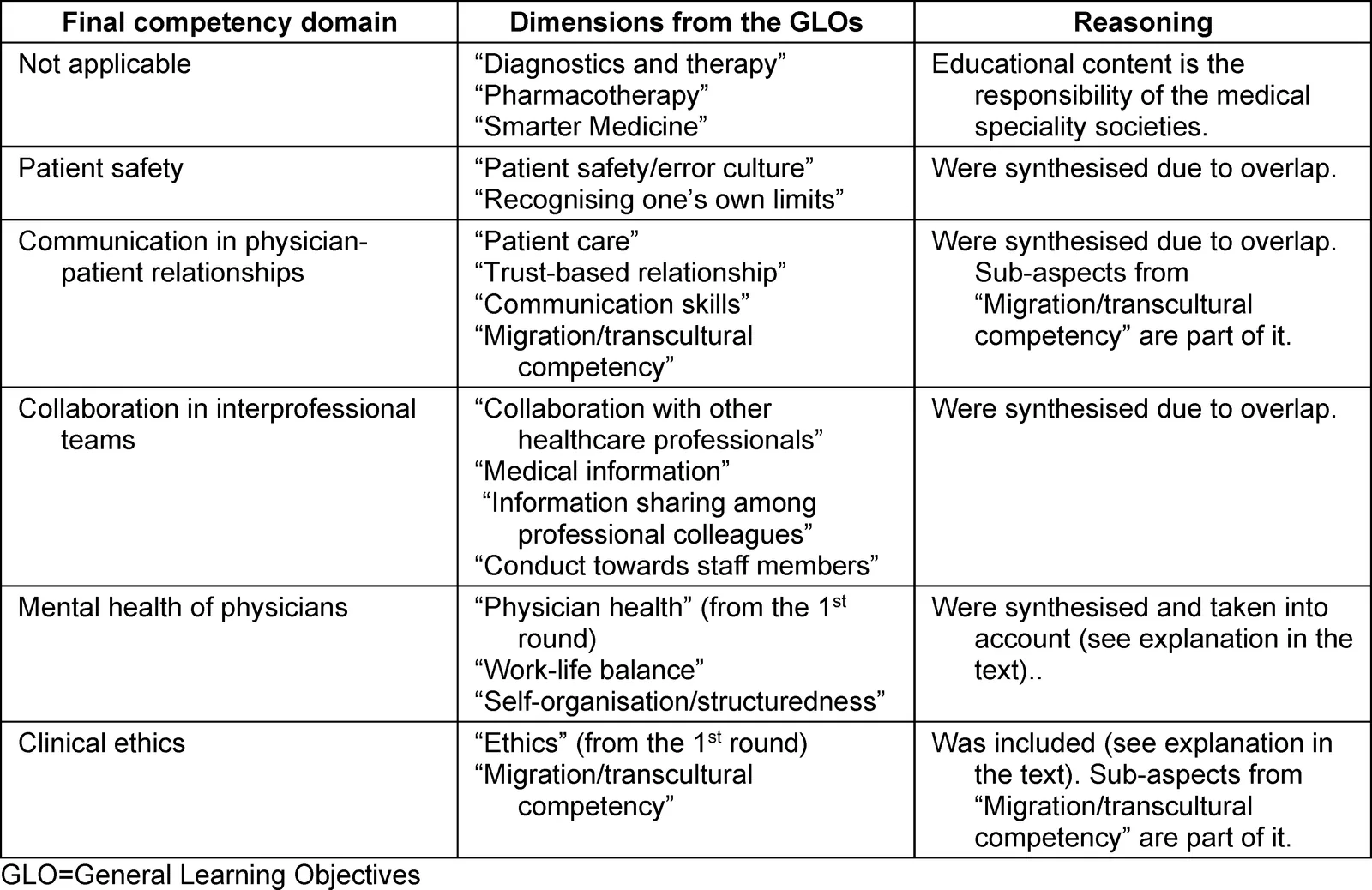
Summarised conceptualisation process within the research team

**Figure 1:**
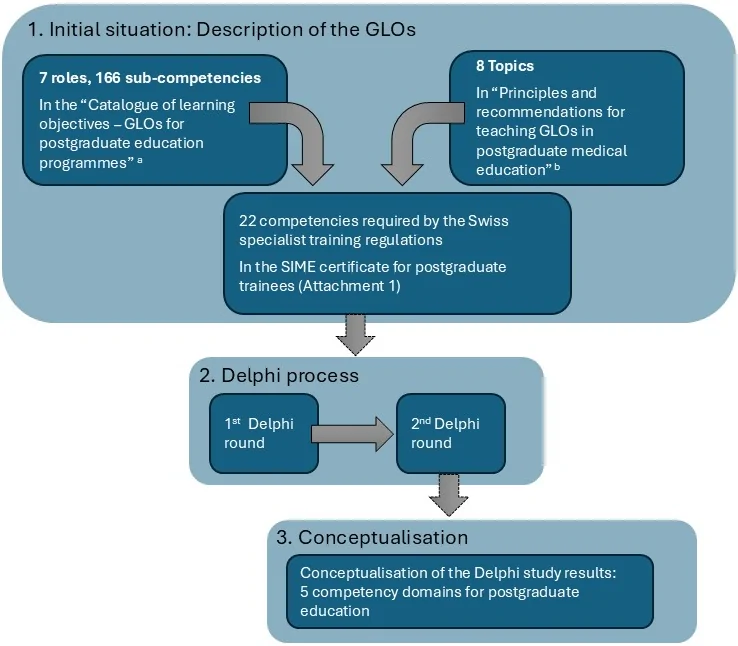
Methodology for prioritising the general learning objectives GLO=General Learning Objectives; SIME= Swiss Institute of Medical Education. ^a^ [2], ^b^ [10]

**Figure 2:**
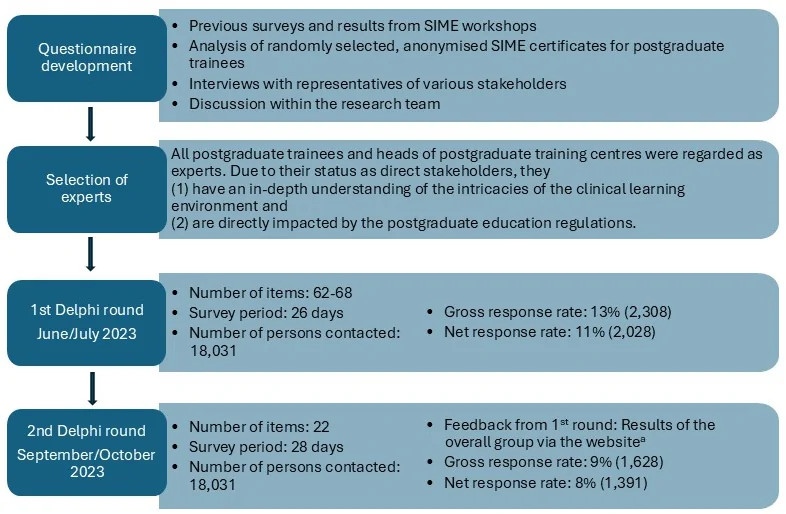
The Delphi process The gross response rate is based on all questionnaires received. The net response rate is based on fully completed questionnaires. ^a^ [16]
