# Supplementary material for: Cross-disciplinary “General Learning Objectives” for postgraduate medical training: Prioritisation of five competency domains using a Delphi process
Source: GMS J Med Educ. 2026 Sep 15;43(6):Doc81. doi: 10.3205/zma001875 (PMC13617337; doi:10.3205/zma001875)
Supplement: Supplementary file 1 [file JME-43-81-s-001.pdf]

**Attachment 1: Certificate (Translation of SIWF-Zeugnis: WBO-Kompetenzen)**

This is an English translation of a questionnaire that is available only in German.

SIWF-Zeugnis

SIWF FMH ISFM

⚠ **Note:** Please fill out this section manually!

Please answer the questions during a joint discussion, taking into account what can be expected of the resident in light of the current level of training or year of residency.

| *Translation only for publication purposes* | Completely true | Somewhat true | Somewhat not true | Not true at all | Not applicable |
|---|---|---|---|---|---|
| **Patient care:**<br>Is able to provide patients with comprehensive advice, support and care, whilst duly respecting their autonomy | | | | | |
| **Trust-based relationship:**<br>Can build a trust-based relationship with patients and their families | | | | | |
| **Communication skills:**<br>Can provide expert information on upcoming diagnostic and therapeutic measures (informed consent discussion) and can deliver bad news in a sensitive and objective manner | | | | | |
| **Diagnostics and therapy:**<br>Can take a concise medical history, carry out a proper clinical examination, use the results to derive a working hypothesis and draw up a plan for the next steps (where possible, in accordance with the principles of evidence-based medicine) | | | | | |
| **Ethics:**<br>Can apply tools for ethical decision-making and independently handle ethical issues that arise in professional routine | | | | | |
| **Health economics:**<br>Considers a reasonable cost-benefit analysis (principle of “effectiveness, appropriateness and cost-effectiveness”) when rendering diagnosis, therapy and prophylaxis | | | | | |

| | | | | | |
|---|---|---|---|---|---|
| **Patient safety/error culture:**<br>Identifies risks, errors and complications at an early stage and manages them appropriately | | | | | |
| **Recognising one's own limits:**<br>Consults other professionals in cases of uncertainty, if the issues lie outside the personal area of expertise, or if the patient requests it | | | | | |
| **Pharmacotherapy:**<br>Knows the indications, pharmacokinetics and relevant adverse effects and interactions of the medicines used | | | | | |
| **Prescribing:**<br>Knows the principles of prescribing and follows the safety guidelines when writing prescriptions | | | | | |
| **Documentation:**<br>Keeps ongoing records on medical history, findings, clinical course and conclusions drawn | | | | | |
| **Medical information:**<br>Maintains confidentiality of medical information | | | | | |
| **Information sharing among professional colleagues:**<br>Summarises relevant information concisely when reporting about a patient | | | | | |
| **Collaboration with other healthcare professionals:**<br>Respects the different roles and responsibilities when collaborating with other healthcare professionals | | | | | |
| **Conduct towards superiors:**<br>Recognises authority and strives to maintain an open, harmonious relationship | | | | | |
| **Conduct towards staff members:**<br>Motivates and manages subordinate staff respectfully | | | | | |
| **Swiss healthcare system:**<br>Knows its structure and organisation | | | | | |
| **Swiss insurance system:**<br>Knows and complies with the requirements of the Swiss insurance system | | | | | |
| **Prevention:**<br>Identifies health problems early and takes appropriate diagnostic and/or preventive measures for individuals and communities | | | | | |
| **Postgraduate medical training and continuous professional education:**<br>Seeks, organises and evaluates medical knowledge from relevant sources | | | | | |

| | | | | | |
|---|---|---|---|---|---|
| **Medical research involving human subjects:**<br>Knows the guidelines of the Swiss Academy of Medical Sciences (SAMW), the provisions of the Federal Constitution of the Swiss Confederation Art. 118b and the provisions of the Swiss Therapeutic Products Act (HMG), Swiss Federal Human Research Act (HRA) and their ordinances, (Ordinance on Clinical Trials [ClinO], Human Research Ordinance [HRO], HRA Organisation Ordinance [OrgO-HRA]) and applies them correctly | | | | | |
| **Self-organisation/structuredness:**<br>Demonstrates appropriate time and task management, sets priorities correctly | | | | | |
