# Supplementary material for: Cross-disciplinary “General Learning Objectives” for postgraduate medical training: Prioritisation of five competency domains using a Delphi process
Source: GMS J Med Educ. 2026 Sep 15;43(6):Doc81. doi: 10.3205/zma001875 (PMC13617337; doi:10.3205/zma001875)
Supplement: Supplementary file 2 [file JME-43-81-s-002.pdf]

Attachment 2: WBO-Kompetenzen und ihre Herleitung aus den Allgemeinen Lernzielen (only in German)

* Lernzielkatalog - Allgemeine Lernziele für die Weiterbildungsprogramme (gemäss Art. 3 Abs. 2 WBO), https://www.siwf.ch/files/pdf29/lernzielkatalog_de.pdf

Nr.	22 WBO-Kompetenz aus E-Logbuch	Lernzielkatalog* (relevante Kompetenz aus Teil A)<br>Der Facharzt ist fähig…	Lernzielkatalog* (relevante Subkompetenz aus Teil B); nur angegeben, wenn Kompetenzen aus Teil A nicht eindeutig
**1**	**Betreuung:**<br>Kann Patientinnen und Patienten umfassend beraten, begleiten und betreuen und dabei deren Autonomie gebührend respektieren	**ME:**… Patienten in Zusammenarbeit mit Angehörigen anderer Berufe zu beraten, zu begleiten und zu betreuen und dabei das Selbstbestimmungsrecht des Patienten gebührend zu respektieren.	
**2**	**Vertrauensbeziehung:**<br>Kann mit Patientinnen und Patienten und Angehörigen eine Vertrauensbeziehung aufbauen	**CM:**… mit Patienten, Familien und weiteren nahestehenden Personen Beziehungen aufzubauen, die von Vertrauen geprägt sind.	
**3**	**Gesprächsführung:**<br>Kann kompetent über bevorstehende diagnostische und therapeutische Massnahmen informieren („Informed Consent“) sowie schlechte Nachrichten einfühlsam und sachlich überbringen	**ME:**… Patienten mit chronischen, nicht heilbaren, progredienten oder weit fortgeschrittenen Erkrankungen über längere Zeiträume angemessen zu betreuen.<br><br>**CM:**… Risiken und Nutzen diagnostischer und therapeutischer Massnahmen individuell verständlich mitzuteilen und damit das informierte Einverständnis („Informed Consent“) zu erreichen.<br><br>**CM:**… einfühlsam schlechte Nachrichten zu überbringen und verantwortungsvoll über Komplikationen und Fehler zu berichten.	**ME 31:** Der Facharzt führt in schwierigen Situationen kompetent Gespräche. Dazu gehören das Überbringen von schlechten Nachrichten, Entscheide über kuratives gegenüber palliatives Vorgehen, die Sterbebegleitung, der Kontakt mit verärgerten oder unkooperativen Patienten sowie das Eingestehen eines eigenen Fehlers.<br><br>**CM 28:** Der Facharzt führt in schwierigen Situationen kompetent Gespräche. Dazu gehören das Überbringen von schlechten Nachrichten, Entscheide über kuratives gegenüber palliatives Vorgehen, die Sterbebegleitung, der Kontakt mit verärgerten oder unkooperativen Patienten sowie das Eingestehen eines eigenen Fehlers.
**4**	**Diagnostik und Therapie:**<br>Kann eine konzise Anamnese aufnehmen, eine korrekte klinische Untersuchung durchführen, daraus eine Arbeitshypothese ableiten und einen Plan zum weiteren Vorgehen aufstellen (wo möglich gemäss den Prinzipien der Evidence-Based Medicine)	**ME:**… die in Anamnese und klinischer Untersuchung erhobenen Informationen zu interpretieren, daraus eine Arbeitshypothese und eine Differentialdiagnose abzuleiten, einen Plan zum weiteren Vorgehen zu erarbeiten und die Erkenntnisse aus der Verlaufsbeobachtung zu nutzen.	

Nr.	22 WBO-Kompetenz aus E-Logbuch	Lernzielkatalog* (relevante Kompetenz aus Teil A)<br>Der Facharzt ist fähig…	Lernzielkatalog* (relevante Subkompetenz aus Teil B); nur angegeben, wenn Kompetenzen aus Teil A nicht eindeutig
**5**	**Ethik:**<br>Kann Instrumente zur ethischen Entscheidungsfindung anwenden und mit ethischen Problemen des beruflichen Alltags selbständig umgehen	**PR:**… zu qualitativ hochstehender Berufsausübung und zeigt dabei eine verantwortungsvolle und empathische Haltung.<br><br>**PR:**… in ethisch verantwortungsbewusster Art zu praktizieren unter Berücksichtigung des Medizinalberu-fegesetzes.	**PR 13:** Der Facharzt setzt sich für Gerechtigkeit in der Gesundheitsversorgung ein<br><br>**PR 17:** Der Facharzt benennt in einer gegebenen klinischen Situation oder in einem Forschungsprojekt die massgebenden ethischen Prinzipien und Werte und wendet die Methoden der ethischen Entscheidungsfindung an. Er berücksichtigt die Einstellung der anderen Beteiligten, begründet seine Haltung und sein eigenes Handeln mit validen Argumenten und übernimmt für sein Handeln die Verantwortung.
**6**	**Gesundheitsökonomie:**<br>Berücksichtigt bei Diagnose, Therapie und Prophylaxe ein vernünftiges Kosten-/ NutzenVerhältnis („WZW-Prinzip“)	**ME:**… alle diagnostischen und therapeutischen Massnahmen unter Berücksichtigung eines vernünftigen Kosten-/Nutzen-Verhältnisses durchzuführen und die Sicherheit der Patienten zu gewährleisten. Dabei soll er das Prinzip „Wirksamkeit-Zweckmässigkeit-Wirtschaftlichkeit“ anwenden.	
**7**	**Patientensicherheit / Fehlerkultur:**<br>Erkennt frühzeitig Risiken, Fehler und Komplikationen und geht angemessen mit ihnen um	**ME:**… Patienten mit chronischen, nicht heilbaren, progredienten oder weit fortgeschrittenen Erkrankungen über längere Zeiträume angemessen zu betreuen<br><br>**ME:**… alle diagnostischen und therapeutischen Massnahmen unter Berücksichtigung eines vernünftigen Kosten-/ Nutzen-Verhältnisses durchzuführen und die Sicherheit der Patienten zu gewährleisten.<br>Dabei soll er das Prinzip „Wirksamkeit-Zweckmässigkeit-Wirtschaftlichkeit“ anwenden.<br><br>**CM:**… einfühlsam schlechte Nachrichten zu überbringen und verantwortungsvoll über Komplikationen und Fehler zu berichten.	**ME 31:** Der Facharzt führt in schwierigen Situationen kompetent Gespräche. Dazu gehören das Überbringen von schlechten Nachrichten, Entscheide über kuratives gegenüber palliatives Vorgehen, die Sterbebegleitung, der Kontakt mit verärgerten oder unkooperativen Patienten sowie das Eingestehen eines eigenen Fehlers.<br><br>**ME 36:** Der Facharzt geht angemessen mit eigenen Fehlern und solchen in seinem Umfeld um. Er lernt aus ihnen und ergreift die notwendigen Massnahmen. (Manager 19).<br><br>**CM 28:** Der Facharzt führt in schwierigen Situationen kompetent Gespräche. Dazu gehören das Überbringen von schlechten Nachrichten, Entscheide über kuratives gegenüber palliatives Vorgehen, die Sterbebegleitung, der Kontakt mit verärgerten oder unkooperativen Patienten sowie das Eingestehen eines eigenen Fehlers.

Nr.	22 WBO-Kompetenz aus E-Logbuch	Lernzielkatalog* (relevante Kompetenz aus Teil A)<br>Der Facharzt ist fähig…	Lernzielkatalog* (relevante Subkompetenz aus Teil B); nur angegeben, wenn Kompetenzen aus Teil A nicht eindeutig
		**MA:**… die Betreuungsqualität und Patientensicherheit zu gewährleisten und zu verbessern	**MA 19:** Der Facharzt geht angemessen mit eigenen Fehlern und solchen in seinem Umfeld um. Er lernt aus ihnen und ergreift die notwendigen Massnahmen.
8	**Erkennen eigener Grenzen:**Zieht andere Fachpersonen bei, wenn sie oder er sich unsicher fühlt, wenn es sich um Probleme ausserhalb der eigenen Fachkompetenz handelt oder wenn die Patientin oder der Patient dies wünscht.	**ME:**… Patienten in Zusammenarbeit mit Angehörigen anderer Berufe zu beraten, zu begleiten und zu betreuen und dabei das Selbstbestimmungsrecht des Patienten gebührend zu respektieren.<br><br>**ME:**… die im Fachbereich üblichen weiterführenden Untersuchungen und Behandlungen durchzuführen und die ausserhalb seiner Fachkompetenz liegenden weiteren Massnahmen zu veranlassen.<br><br>**CO:**… mit anderen Fachärzten und Fachpersonen anderer Berufsgruppen zusammen zu arbeiten, auch bei unterschiedlicher kultureller Herkunft.<br><br>**MA:**… die Betreuungsqualität und Patientensicherheit zu gewährleisten und zu verbessern	**ME 13:** Der Facharzt zieht andere Fachpersonen bei, wenn er sich unsicher fühlt oder wenn der Patient dies wünscht<br><br>**ME 28:** Der Facharzt beachtet seine fachlichen Grenzen und führt Untersuchungen und Behandlungen entsprechend seinen erworbenen Kompetenzen durch.<br><br>**ME 29/ CO 2/ MA 20:** Der Facharzt zieht bei Bedarf andere Fachleute zur Betreuung der Patienten bei.
9	**Pharmakotherapie:**<br>Kennt die Indikationen, die Pharmakokinetik und die relevanten unerwünschten Neben- und Wechselwirkungen der eingesetzten Arzneimittel	**ME**:… alle diagnostischen und therapeutischen Massnahmen unter Berücksichtigung eines vernünftigen Kosten-/Nutzen-Verhältnisses durchzuführen und die Sicherheit der Patienten zu gewährleisten. Dabei soll er das Prinzip „Wirksamkeit-Zweckmässigkeit-Wirtschaftlichkeit“ anwenden.	**ME 38:** Der Facharzt beschreibt die Indikationen, die Pharmakokinetik und die klinisch relevanten unerwünschten Wirkungen der eingesetzten Arzneimittel inkl. Diagnostica wie Kontrastmittel. Er verringert mögliche Wechselwirkungen und berücksichtigt bei der Dosierung das Alter und relevante Organinsuffizienzen, indem er geeignete elektronische Hilfsmittel verwendet.<br><br>**ME 39:** Der Facharzt wendet Arzneimittel und Medizinprodukte zweckmässig an und berücksichtigt dabei die „WZW-Kriterien“.
10	**Rezeptieren:**<br>Kennt die Prinzipien des Rezeptierens und beachtet die Sicherheitsprinzipien bei der Ausstellung von Rezepten	**ME**:… alle diagnostischen und therapeutischen Massnahmen unter Berücksichtigung eines vernünftigen Kosten-/Nutzen-Verhältnisses durchzuführen und die Sicherheit der Patienten zu gewährleisten. Dabei soll er das Prinzip „Wirksamkeit-Zweckmässigkeit-Wirtschaftlichkeit“ anwenden.	**ME 45:** Der Facharzt beachtet die Sicherheitsprinzipien bei der Ausstellung von Rezepten.

Nr.	22 WBO-Kompetenz aus E-Logbuch	**Lernzielkatalog* (relevante Kompetenz aus Teil A)** Der Facharzt ist fähig…	**Lernzielkatalog* (relevante Subkompetenz aus Teil B); nur angegeben, wenn Kompetenzen aus Teil A nicht eindeutig**
**11**	**Dokumentation:**<br>Dokumentiert fortlaufend Anamnese, Befunde und den Krankheitsverlauf sowie die daraus abgeleiteten Folgerungen	**ME:**… die medizinischen Informationen geeignet festzuhalten und zu schützen.<br><br>**CM:**… die Informationen aus einer fachärztlichen Konsultation / Visite zu dokumentieren und zeitgerecht weiterzugeben.	**ME 50:** Der Facharzt dokumentiert seine Befunde aus Anamnese, Status und zusätzlichen Untersuchungen sowie den Behandlungsplan auf verständliche und sachdienliche Art.<br><br>**CM 25:** Der Facharzt dokumentiert seine Befunde aus Anamnese, Status und zusätzlichen Untersuchungen sowie den Behandlungsplan auf verständliche und sachdienliche Art.
**12**	**Medizinische Information:**<br>Beachtet die Vertraulichkeit medizinischer Information	**ME:**… Patienten in Zusammenarbeit mit Angehörigen anderer Berufe zu beraten, zu begleiten und zu betreuen und dabei das Selbstbestimmungsrecht des Patienten gebührend zu respektieren.	**ME 18:** Der Facharzt beachtet die Vertraulichkeit medizinischer Informationen.
**13**	**Information von Berufskolleginnen und Berufskollegen:**<br>Fasst die relevanten Daten konzis zusammen, wenn sie oder er über eine Patientin oder einen Patienten informiert	**ME:**… Patienten in Zusammenarbeit mit Angehörigen anderer Berufe zu beraten, zu begleiten und zu betreuen und dabei das Selbstbestimmungsrecht des Patienten gebührend zu respektieren.<br><br>**ME:**… die medizinischen Informationen geeignet festzuhalten und zu schützen.<br><br>**CM:**… die wichtigen Informationen von den Patienten und der beteiligten Personen aus deren Umfeld zu gewinnen, zu verarbeiten und die gewonnen Erkenntnisse personen- und situationsgerecht zu kommunizieren.<br><br>**CM:**… die Informationen aus einer fachärztlichen Konsultation / Visite zu dokumentieren und zeitgerecht weiterzugeben.<br><br>**CO:**... mit anderen Fachärzten und Fachpersonen anderer Berufsgruppen zusammen zu arbeiten, auch bei unterschiedlicher kultureller Herkunft.	**ME 8:** Der Facharzt informiert den Patienten und dessen Umfeld sowie weitere involvierte Fachleute und fördert die gemeinsame Entscheidungsfindung.<br><br>**ME 49:** Der Facharzt berichtet konzis über die Befunde und die Vorschläge zum Behandlungsplan. Er erarbeitet die Behandlungsoptionen mit anderen Fachpersonen, bespricht die Evidenzbasis<br>und erwägt möglichen Nutzen und Risiken. (Communicator 24, Collaborator 1<br><br>**CM 12:** Der Facharzt informiert den Patienten und dessen Umfeld sowie weitere<br>involvierte Fachleute und fördert die gemeinsame Entscheidungsfindung.<br><br>**CM 24/ CO 1:** Der Facharzt fasst die relevanten Informationen konzis zusammen, wenn er über seine Befunde und seine Vorschläge zum Behandlungsplan berichtet. Er erarbeitet mit anderen Fachpersonen die Behandlungsoptionen, bespricht die Evidenz-basis und erwägt möglichen Nutzen und Risiken.

Nr.	22 WBO-Kompetenz aus E-Logbuch	Lernzielkatalog* (relevante Kompetenz aus Teil A)<br>Der Facharzt ist fähig…	Lernzielkatalog* (relevante Subkompetenz aus Teil B); nur angegeben, wenn Kompetenzen aus Teil A nicht eindeutig
**14**	**Zusammenarbeit mit anderen Gesundheitsberufen:**<br>Respektiert die unterschiedlichen Aufgaben und Kompetenzen in der Zusammenarbeit mit anderen Gesundheitsberufen	**CO:**… mit anderen Fachärzten und Fachpersonen anderer Berufsgruppen zusammen zu arbeiten, auch bei unterschiedlicher kultureller Herkunft.<br><br>**MA:**… seine Berufstätigkeit erfolgreich zu gestalten und die seiner beruflichen Stellung entsprechenden Management-Aufgaben zu übernehmen.	**CM 24/ CO 1:** Der Facharzt fasst die relevanten Informationen konzis zusammen, wenn er über seine Befunde und seine Vorschläge zum Behandlungsplan berichtet. Er erarbeitet mit anderen Fachpersonen die Behandlungsoptionen, bespricht die Evidenz-basis und erwägt möglichen Nutzen und Risiken.<br><br>**CO 6:** Der Facharzt erläutert die Aufgaben und Funktionen der verschiedenen Berufe und Institutionen im schweizerischen Gesundheitswesen. Er respektiert die unterschiedlichen Aufgaben<br>und Funktionen in der Zusammenarbeit mit Teammitgliedern und Partnern aus verschiedenen Gesundheitsberufen und Institutionen.<br><br>**MA 1:** Der Facharzt erläutert die Aufgaben und Funktionen der verschiedenen Berufe und Institutionen im schweizerischen Gesundheitswesen. Er respektiert die unterschiedlichen Aufgaben und Funktionen in der Zusammenarbeit mit Teammitgliedern und Partnern aus verschiedenen Gesundheitsberufen und Institutionen.
**15**	**Verhalten gegenüber Vorgesetzten:**Anerkennt Autorität, bemüht sich um eine offene, harmonische Beziehung	**CO:**… Interessensgegensätze zu erkennen, Meinungen Anderer zu akzeptieren und in der Zusammenarbeit Konflikte zu vermeiden und zu lösen.	**CO 13:** Der Facharzt pflegt einen respektvollen Umgang mit Mitarbeitern und Arbeitspartnern. Er trägt zu einer vertrauensvollen, offenen Atmosphäre bei und respektiert unterschiedliche Standpunkte.**CO 14:** Der Facharzt beschreibt die Zusammenarbeit im Team und die Teamdynamik und erkennt Missverständnisse, Spannungen und Leistungsgrenzen bei sich und den anderen Teammitgliedern. Er verhindert Konflikte. Er trägt zur Lösung bestehender Konflikte bei.
**16**	**Verhalten gegenüber Mitarbeitenden:**<br>Motiviert und führt die ihr oder ihm unterstellten mitarbeitenden respektvoll	**CO:**… Interessensgegensätze zu erkennen, Meinungen Anderer zu akzeptieren und in der Zusammenarbeit Konflikte zu vermeiden und zu lösen.	**CO 13:** Der Facharzt pflegt einen respektvollen Umgang mit Mitarbeitern und Arbeitspartnern. Er trägt zu einer vertrauensvollen, offenen Atmosphäre bei und respektiert unterschiedliche Standpunkte.<br><br>**CO 14:** Der Facharzt beschreibt die Zusammenarbeit im Team und die Teamdynamik und erkennt Missverständnisse, Spannungen und Leistungsgrenzen bei sich und den anderen Teammitgliedern. Er verhindert Konflikte. Er trägt zur Lösung bestehender Konflikte bei.

Nr.	22 WBO-Kompetenz aus E-Logbuch	Lernzielkatalog* (relevante Kompetenz aus Teil A)<br>Der Facharzt ist fähig…	Lernzielkatalog* (relevante Subkompetenz aus Teil B); nur angegeben, wenn Kompetenzen aus Teil A nicht eindeutig
17	**Schweizerisches Gesundheitswesen:**<br>Kennt dessen Struktur und Organisation	**ME:** … die Gesundheit von Patienten und der Gemeinschaft zu fördern.<br><br>**MA:**… die beschränkten Mittel im Gesundheitswesen effizient im besten Interesse der Patienten unter Berücksichtigung der Wirksamkeit, Zweckmässigkeit und Wirtschaftlichkeit einzusetzen.<br><br>**MA:**… die Betreuungsqualität und Patientensicherheit zu gewährleisten und zu verbessern<br><br>**HA:**… die massgebenden Einflussfaktoren auf die Gesundheit von Einzelnen und eines Kollektivs zu beschreiben und zur Erhaltung und Verbesserung der Gesundheit beizutragen.<br><br>**PR:**… zu qualitativ hochstehender Berufsausübung und zeigt dabei eine verantwortungsvolle und empathische Haltung.	**ME 4:** Der Facharzt empfiehlt Screening-Untersuchungen und Impfungen entsprechend den allgemein anerkannten Richtlinien und nationalen Vorschriften.<br><br>**MA 9:** Der Facharzt bemüht sich um eine gerechte Verwendung der Ressourcen im Gesundheitswesen. Er erkennt Konflikte zwischen den Bedürfnissen einzelner Patienten und der Gemeinschaft.<br><br>**MA 11:** Der Facharzt kennt und beachtet bei seiner täglichen Arbeit die Vorgaben des Schweizerischen Gesundheits- und Sozialversicherungssystems<br><br>**MA 18:** Der Facharzt erkennt gefährliche Situationen und sorgt für die nötigen Massnahmen.<br><br>**HA 5:** (Medical Expert 4) Der Facharzt empfiehlt Screening-Untersuchungen und Impfungen entsprechend den allgemein anerkannten Richtlinien und nationalen Vorschriften.<br><br>**HA 6:** Der Facharzt erfüllt die gesetzliche Meldepflicht von Krankheiten.<br><br>**HA 7:** Der Facharzt bemüht sich um eine gerechte Verwendung der Ressourcen im Gesundheitswesen. Es ist ihm bewusst, dass Konflikte zwischen den Bedürfnissen einzelner Patienten und der Gemeinschaft bestehen können.<br><br>**PR 13:** Der Facharzt setzt sich für Gerechtigkeit in der Gesundheitsversorgung ein

Nr.	22 WBO-Kompetenz aus E-Logbuch	**Lernzielkatalog* (relevante Kompetenz aus Teil A)** Der Facharzt ist fähig…	**Lernzielkatalog* (relevante Subkompetenz aus Teil B); nur angegeben, wenn Kompetenzen aus Teil A nicht eindeutig**
**18**	**Schweizerisches Versicherungssystem:** Kennt und beachtet die Vorgaben des Schweizerischen Versicherungssystems	**MA:**… die beschränkten Mittel im Gesundheitswesen effizient im besten Interesse der Patienten unter Berücksichtigung der Wirksamkeit, Zweckmässigkeit und Wirtschaftlichkeit einzusetzen.<br><br>**PR:**… in ethisch verantwortungsbewusster Art zu praktizieren unter Berücksichtigung des Medizinalberufegesetzes.	**MA 11:** Der Facharzt kennt und beachtet bei seiner täglichen Arbeit die Vorgaben des Schweizerischen Gesundheits- und Sozialversicherungssystems<br><br>**PR 15:** Der Facharzt nennt und beschreibt die für sein Fachgebiet und die Art seiner beruflichen Tätigkeit massgebenden Inhalte der kantonalen und schweizerischen Gesetze und Verordnungen. Dazu gehören insbesondere Vorschriften über Patientenverfügungen, ärztliche Zeugnis gen. Dazu gehören insbesondere Vorschriften über Patientenverfügungen, ärztliche Zeugnisse, Feststellung des Todes und die Arzneimittelverschreibung (Heilmittelgesetz, Betäubungsmittelgesetz, Krankenversicherungsgesetz). Er erfüllt die entsprechenden Verpflichtungen und kennt die Konsequenzen, wenn er dagegen verstösst.
**19**	**Prävention:** Erkennt Gesundheitsprobleme frühzeitig und ergreift adäquate diagnostische und/oder präventive Massnahmen bei Einzelpersonen und Kollektiven	**HA:**… Gesundheitsprobleme zu erkennen und die notwendigen Massnahmen zu ergreifen.	
**20**	**Weiter- und Fortbildung:** Sucht, ordnet und wertet medizinisches Wissen aus den relevanten Quellen	**ME:**… medizinische Kompetenzen zu erwerben zu erhalten und zu erweitern.<br><br>**MA:**… relevante Informationen zur Patientenbetreuung zu werten und zu nutzen.<br><br>**SC:**… sich auf seine Berufstätigkeit ausgerichtet ständig fortzubilden.<br><br>**SC:**… medizinische Fachinformation und deren Quellen zu evaluieren und in seinen Entscheiden zu berücksichtigen.	**ME 54:** Der Facharzt lernt ständig, indem er medizinisches Wissen aus den relevanten Quellen sucht, sammelt, ordnet und wertet. Er wendet dieses Wissen bei der Patientenbetreuung kritisch an.<br><br>**MA 14:** Der Facharzt lernt ständig, indem er medizinisches Wissen aus den relevanten Quellen sucht, sammelt, ordnet und wertet (Weiter- und Fortbildung). Er wendet dieses Wissen bei der Patientenbetreuung kritisch an

Nr.	22 WBO-Kompetenz aus E-Logbuch	Lernzielkatalog* (relevante Kompetenz aus Teil A)<br>Der Facharzt ist fähig…	Lernzielkatalog* (relevante Subkompetenz aus Teil B); nur angegeben, wenn Kompetenzen aus Teil A nicht eindeutig
**21**	**Forschung am Menschen:**<br>Kennt die Richtlinien der SAMW (Schweizerische Akademie medizinischer Wissenschaften), die Bestimmungen der Bundesverfassung Art. 118b und die Bestimmungen von HMG (Heilmittelgesetz), HFG (Humanforschungsgesetz) und deren Verordnungen (Verordnung über klinische Versuche, Verordnung über nicht als klinische Versuche geltende Projekte der Forschung am Menschen, Organisationsverordnung zum Humanforschungsgesetz) und wendet sie korrekt an	**SC:**… medizinische Fachinformation und deren Quellen zu evaluieren und in seinen Entscheiden zu berücksichtigen.<br><br>**SC:**… zur Entwicklung, Verbreitung und Umsetzung von neuen Erkenntnissen und Methoden beizutragen.	**SC 4:** Der Facharzt beschreibt grundlegende Methoden und Prinzipien der medizinischen Forschung. Er kennt insbesondere das Humanforschungsgesetz.
**22**	**Selbstorganisation/ Strukturiertheit:**<br>Zeigt adäquates Zeit- und Aufgabenmanagement, setzt richtige Prioritäten	**MA:...** ein Gleichgewicht zu finden zwischen seiner Berufstätigkeit und seinen privaten Aktivitäten.	
	**Allgemeine Lernziele ohne direkte Entsprechung zu den WBO-Kompetenzen**	**ME:**… eine konzise Anamnese zu erheben.	
		**ME:**… eine Untersuchung («klinische Untersuchung») durchzuführen.	
		**CM:**… auf die Befriedigung von Eigeninteressen, insbesondere auf jede Form sexueller Kontakte zu den ihm anvertrauten Patienten zu verzichten.	
		**CM:**… diagnostische und therapeutische Entscheidungen, die nicht urteils- bzw. handlungsfähige Patienten betreffen, gemeinsam mit den zuständigen Vertretern zu fällen.	
		**SC:**… Patienten, Medizinstudenten, andere Ärzte, Angehörige von Behörden und weitere an der Gesundheitsversorgung beteiligte Personen zu unterrichten und beim Lernen zu unterstützen.	

Abkürzungen: ME=Medical Expert; CM=Communicator; CO=Collaborator; MA=Manager; HA=Health Advocate; SC=Scholar; PR=Professional

Attachment 2 to Bayha E, Armstad H, Spinas G, Brodmann Maeder M, Hennel EK. *Cross-disciplinary "General Learning Objectives" for postgraduate medical training: Prioritisation fo five competency domains using a Delphi prozess*. GMS J Med Educ. 2026;43(6):Doc81. DOI: 10.3205/zma001875
