# Supplementary material for: Cross-disciplinary “General Learning Objectives” for postgraduate medical training: Prioritisation of five competency domains using a Delphi process
Source: GMS J Med Educ. 2026 Sep 15;43(6):Doc81. doi: 10.3205/zma001875 (PMC13617337; doi:10.3205/zma001875)
Supplement: Supplementary file 3 [file JME-43-81-s-003.pdf]

Attachment 3: Fragebogen Delphistar (only in German)

Attachment 3 to Bayha E, Armstad H, Spinas G, Brodmann Maeder M, Hennel EK. *Cross-disciplinary "General Learning Objectives" for postgraduate medical training: Prioritisation fo five competency domains using a Delphi prozess*. GMS J Med Educ. 2026;43(6):Doc81. DOI: 10.3205/zma001875

Delphi studies in social and health sciences – recommendations for an interdisciplinary standardized reporting (DELPHISTAR). *Delphi-Studien in den Sozial- und Gesundheitswissenschaften - Empfehlungen für eine interdisziplinäre, standardisierte Berichterstattung*

Von: Niederberger, M., Schifano, J., Deckert, S., Hirt, J., Homberg, A., Köberich, S., Kuhn, R., Rommel, A., Sonnberger, M. & the DEWISS network (2024). Delphi studies in social and health sciences—Recommendations for an interdisciplinary standardized reporting (DELPHISTAR). Results of a Delphi study. *PLoS ONE 19(8):* e0304651. https://doi.org/10.1371/journal.pone.0304651

Mehr Informationen unter OSF (https://osf.io/gc4jk) und DEWISS (https://delphi.ph-gmuend.de/)

Was ist das Ziel von DELPHISTAR?

- Berichterstattung über Delphi-Studien verbessern, vereinheitlichen und vergleichbar machen
- Erleichterung der Bewertung von Delphi-Studien, auch im Rahmen von Peer-Review-Verfahren
- Inkonsistenzen und unklare Beschreibungen in Publikationen über Delphi-Studien reduzieren und idealerweise verhindern
- Bewusstsein für die Vielfalt der Delphi-Varianten und ihre spezifischen Potenziale bzw. Herausforderungen schärfen

DELPHISTAR ist eine Delphi Reporting Guideline, die:

- für alle Delphi-Varianten (z. B. klassisches Delphi, Real-Time Delphi, Gruppendelphi, Policy Delphi, Argumentatives Delphi, Café Delphi) gültig ist
- für unterschiedliche Zwecke anwendbar ist (z. B. Delphi-Studien zur Konsensfindung, zur Sammlung von Expert*innenurteilen oder für Prognosen)
- in den Gesundheits- und Sozialwissenschaften gleichermaßen berücksichtigt wird

Diese Reporting Guideline gilt für Studien aus den Gesundheits- und Sozialwissenschaften, die Delphi-Verfahren nutzen. Hierbei sind alle Delphi-Varianten und Modifikationen inbegriffen, die folgende Kriterien erfüllen**:**

1. Befragung mehrerer Personen (=Expert*innen) mit spezialisiertem Wissen (z. B. operatives Wissen, Erfahrungswissen, funktionales Wissen, kontextbezogenes Wissen)
2. Strukturierter Kommunikationsprozess
3. Durchführung von mindestens zwei Erhebungsrunden oder die Möglichkeit, mindestens zwei Mal zu antworten
4. Feedback: Rückmeldung der (Zwischen-)Ergebnisse ab der zweiten Runde
5. Grundlage ist ein quantitativer Fragebogen mit der Möglichkeit, offen Argumente für die jeweilige Position einzubringen oder zu ergänzen
6. Quantitative und qualitative Antworten werden systematisch analysiert (quantitativ: z. B. deskriptive Statistik, qualitativ: z. B. thematische Analyse)

Diese Reporting Guideline ist auf Englisch und Deutsch verfügbar unter https://delphi.ph-gmuend.de/activities/delphistar (zuletzt überarbeitet im Oktober 2024).

Kontakt

Prof. Dr. Marlen Niederberger
E-Mail: marlen.niederberger@ph-gmuend.de
Abteilung Forschungsmethoden für Gesundheitsförderung und Prävention
Institut für Gesundheitswissenschaften, Pädagogische Hochschule Schwäbisch Gmünd
Oberbettringer Straße 200, 73525 Schwäbisch Gmünd, Deutschland

Thema	Sektion	Item	Checklist Item	Item erwähnt auf Seite	Formulierung oder Bemerkung
I Titel und Abstract		1	Identifikation als Delphi-Verfahren im Titel	Seite 1, Zeile 1-5	Die fachübergreifenden «Allgemeinen Lernziele» für die ärztliche Weiterbildung: Priorisierung von fünf Kompetenzbereichen mithilfe eines Delphi-Verfahrens
		2	Identifikation als Delphi-Verfahren im Abstract	Seite 2, Zeile 22-25	„In einem Delphi-Verfahren wurden Ärzt*innen in Weiterbildung…“
		3	Strukturierung des Abstracts	Seite 2	Zielsetzung, Methodik, Ergebnisse und Schlussfolgerung
II Kontext	Formales	4	Informationen zu Geldgeber*innen/Förderung der Studie	-	Es gab keine externe Finanzierung
		5	Informationen zum Autor*innenteam und/oder die Forscher*innen (z. B. Fachgebiet, Institution)	Seite 8, Pkt 2.4	2.4 „Forscherteam und Studienbeitrag“
		6	Informationen über eine Methodenberatung	-	Es fand keine methodische Beratung statt.
		7	Informationen zum Projekthintergrund	Seite 3, 76-79; Seite 4, Abb 1	„Diese Studie untersucht die Umsetzung und inhaltliche Gewichtung der bestehenden ALZ bei Ärzt*innen in Weiterbildung und Leiter*innen von WBS, um eine praktikable Anzahl von bedarfsgerechten fachübergreifenden Kompetenzen für die ärztliche Weiterbildung zu formulieren.“ „Abb.1 Methodisches Vorgehen bei der Evaluation der ALZ“
		8	Informationen zum Studienprotokoll	-	Das Studienprotokoll wurde nicht veröffentlicht.
	Inhalte	9	Begründung der gewählten Methode (Delphi-Verfahren) für die Beantwortung der Forschungsfrage	Punkt 4.5 Stärken und Grenzen, Seite16	„Delphi-Verfahren sind eine geeignete Methode, Expertenmeinungen einzuholen, um Interventionen zu planen.“ Der Fokus unseres Delphi Verfahrens liegt auf der quantifizierenden Bewertung der Expert*innen, weil der Sachverhalt auf den bestehenden WBO-Kompetenzen basiert. Neue Themen werden als Ergänzung abgefragt. Siehe auch [M. Häder. Delphi-Befragungen. Ein Arbeitsbuch. Springer (2014)]
		10	Ziel des Delphi-Verfahrens (z. B. Konsens, Vorhersage)	Seite 5, Zeilen 100-103	„Mit dem Ziel, einen Konsens über die Relevanz bestehender ALZ und möglicher neuer Themen zu finden, möchten wir die Evidenz und Akzeptanz notwendiger, fachübergreifender Bildungsstandards für Ärzt*innen in Weiterbildung verbessern.“

Attachment 3 to Bayha E, Armstad H, Spinas G, Brodmann Maeder M, Hennel EK. *Cross-disciplinary "General Learning Objectives" for postgraduate medical training: Prioritisation fo five competency domains using a Delphi prozess*. GMS J Med Educ. 2026;43(6):Doc81. DOI: 10.3205/zma001875

Thema	Sektion	Item	Checklist Item	Item erwähnt auf Seite	Formulierung oder Bemerkung
III Methode	Aufbau und Integration von Wissen	11	Identifizierung und Begründung relevanter Expertise, Erfahrungsbereiche und Perspektiven (z. B. Theorie, Praxis, betroffene Gruppen, Disziplinen)	Seite 5, Durch-führung	„Als primär Beteiligte werden alle Schweizer Ärzt*innen in Weiterbildung und Leiter*innen von WBS als Expert*innen angesehen." &
		12	Umgang mit fehlendem oder bewusst nicht integriertem Wissen, Expertise und Perspektiven	-	Es gab keine Perspektive, die beabsichtigt nicht inkludiert wurde.
		13	Grundlegende Definition des Begriffs Expert*in[1]	Seite 7, Abb 2	„Als Kontextexpert*innen werden alle Ärzt*innen in Weiterbildung und Leiter*innen von Weiterbildungsstätten angesehen, da sie als unmittelbar Beteiligte (1) einen vertieften Einblick in die Gegebenheiten der klinischen Lernumgebung haben und (2) von den Weiterbildungsvorgaben unmittelbar betroffen sind."
	Delphi-Variante und Modifika-tionen	14	Nennung der Delphi-Variante und möglicher Modifikationen (z. B. klassisches Delphi, Real-Time-Delphi, Gruppendelphi)	-	Eingesetzt wird ein modifiziertes Delphi-Verfahren mit einer Totalerhebung der Expertengruppe. Sie ähnelt der Typ 3 Delphibefragung „Ermittlung von Expertenmeinungen": „Resultate solcher Studien dienen beispielsweise dazu, um gezielte Schlussfolgerungen für erforderliche Interventionen zu ziehen, um anstehende Entscheidungen abzuleiten, um eine Sensibilisierung gegenüber Fehlentwicklungen zu erreichen oder um diese gegebenenfalls zu korrigieren.»; «Experten werden über Totalerhebung oder bewusste Auswahl rekrutiert; je mehr Befragte, desto aussagekräftiger wird das Ergebnis.» [M. Häder. Delphi-Befragungen. Ein Arbeitsbuch. Springer (2014)]
		15	Begründung der Delphi-Variante und der Modifikationen, ggf. auch während des Delphi-Verfahrens	Siehe #14 & Seite 5, Zeile 128/ 129	Siehe #14 & „Aus Gründen der Teilnahmebereitschaft wurde auf eine dritte Runde verzichtet."
	Stichprobe der Expert*innen	16	Auswahlkriterien für die Expert*innen (pro Runde, ggfs. pro Expert*innengruppen)	Seite 7, Abb 2 & Seite 5, Durch-führung	Siehe #13 & „Zum Zeitpunkt der Umfragen umfasste dies 14'130 Ärzt*innen in Weiterbildung und 3'901 Leiter*innen von WBS."
		17	Identifikation der Expert*innen	-	Über die Nutzung des SIWF E-Logbuchs, dass für die Facharzttitelvergabe obligatorisch zu führen ist, werden die Ärzt*innen in Weiterbildung identifiziert. Im Akkreditierungsverfahren für Weiterbildungsstätten werden die Leiter*innen der WBS als

Thema	Sektion	Item	Checklist Item	Item erwähnt auf Seite	Formulierung oder Bemerkung
					Weiterbildungsverantwortliche benannt und sind dem SIWF bekannt
		18	Informationen zur Rekrutierung und eventueller Nachrekrutierung von Expert*innen	-	Die Stichproben der ersten und zweiten Umfrage unterscheiden sich. Durch die Totalerhebung war es möglich an der zweiten Umfrage teilzunehmen und bei der ersten nicht. Das war bei 38.7% der Fall (Tabelle 1). Bezüglich der soziodemographischen Charakteristika unterscheiden sich die beiden Umfragen-Stichproben nur geringfügig (Tabelle 1), deshalb gehen wir dennoch von einem konsistenten Umfrageergebnis aus.
	**Fragebogen/ Erhebungs-instrument**	19	Erläuterung der Entwicklung des Fragebogens[2]	Seite 5; Zeile 105-108	Der Fragebogen wurde auf der Basis früherer Umfragen und Workshops des SIWF, auf der Analyse zufällig ausgewählter E-Logbücher sowie auf Grundlage von Interviews mit Vertreter*innen verschiedener Interessensgruppen entwickelt. Eine Testversion wurde im Forscherteam diskutiert und adaptiert.
		20	Beschreibung des Fragebogens (Inhalt und Aufbau)	Seite 5 Frage-bogen; Anhang 4 & 5	„Der Fragebogen enthielt Fragen zu…“
	**Delphi-Runden**	21	Anzahl der Delphi-Runden	Seite 7, Abb 2	Zwei Delphi-Umfragen
		22	Informationen über die Ziele der einzelnen Delphi-Runden	Seite 5, Zeile 100-103; Seite 3, 79-81; Seite 10, Zeile 215/216	**Allgemein:** „Mit dem Ziel einen Konsens über die Relevanz bestehender ALZ und möglicher neuer Themen zu finden“; **Erste Umfrage:** Information darüber, warum die ALZ zu wenig Beachtung finden und welche ALZ als sehr relevant eingestuft werden (Konsens bei >= 60%). FF 1 & FF 2 **Zweite Umfrage:** „das Ergebnis der ersten Umfrage validiert und die Anzahl der Kompetenzen reduziert werden“
		23	Offenlegung und Begründung des Kriteriums für die Beendigung des Delphi-Verfahrens	Seite 6, Zeile 112/ 113	„Aus Gründen der Teilnahmebereitschaft wurde auf eine dritte Runde verzichtet.“
	**Feedback**	24	Informationen darüber, welche Daten pro Runde rückgemeldet wurden	Seite 5, Durch-führung; Seite 7, Abb 2	„Im E-Mailtext zur zweiten Umfrage wurde via Link auf die Ergebnisse der ersten Umfrage verwiesen, die auf der Webseite des SIWF veröffentlicht wurden“
		25	Informationen darüber, wie die Ergebnisse der vorangegangenen Delphi-Runde an die befragten Expert*innen zurückgemeldet wurden (z. B.	-	Siehe #24

Thema	Sektion	Item	Checklist Item	Item erwähnt auf Seite	Formulierung oder Bemerkung
			über Häufigkeiten, Mittelwerte, Streuungsmaße, Auflistung der Kommentare)		
		26	Informationen darüber, ob das Feedback nach bestimmten Gruppen differenziert wurde (z. B. nach Fachgebiet, institutioneller Zugehörigkeit)	-	Siehe #24
		27	Informationen darüber, wie mit Dissens und unklaren Ergebnissen umgegangen wurde	Seite 8, Pkt. 2.3; Seite 13, Tabelle 4	Konzeptualisierung im Forscherteam
	**Datenanalyse**	28	Offenlegung der quantitativen und qualitativen Auswertungsstrategie	Seite 5/6, Frage-bogen, Daten-analyse	„Neue Themen werden inhaltlich zusammengefasst“ „Die statistische Analyse wurde in Excel durchgeführt. Die Antworten auf die standardisierten Fragen wurden deskriptiv ausgewertet.“
		29	Definition und Messung von Konsens	Seite 5/6, Frage-bogen	„Ein Konsens für ein Item besteht, wenn mindestens 60% der Antwortenden das Item als „sehr relevant” bewerten. Neue Themen werden inhaltlich zusammengefasst und berücksichtigt, wenn sie insgesamt mehr als 20-mal genannt werden.“ „Die acht am höchsten bewerteten Items sollen in der Weiterbildung berücksichtigt werden“
		30	Angaben zur gruppenspezifischen Analyse oder Gewichtung der Expert*innen (z.B. Theorie versus Praxis, disziplinspezifische Analyse)	Seite 11	Tabelle 3
**IV Ergebnisse**	**Delphi-Prozess**	31	Veranschaulichung des Delphi-Verfahrens (z. B. in einem Flussdiagramm)	Seite 5; 7	Abb1 & Abb 2
		32	Informationen über besondere Aspekte während des Delphi-Verfahrens (z. B. Abweichungen von der geplanten Vorgehensweise mit Begründung)	Seite 12, ab Zeile 230; Seite 13, Tabelle 4	„Bei der Durchsicht der Ergebnisse fielen folgende formalen Widersprüche auf…“
		33	Anzahl der Expert*innen pro Runde (sowohl eingeladene als auch teilnehmende)	Seite 5, Durch-führung; Seite 9, Tabelle 1	„Zum Zeitpunkt der Umfragen umfasste dies 14.130 Ärzt*innen in Weiterbildung und 3.901 Leiter*innen von WBS.“; „An der ersten Umfrage nahmen insgesamt 13% (n=2.308), an der zweiten Umfrage 9% (n=1.628) der angeschriebenen Ärzt*innen teil.“

Thema	Sektion	Item	Checklist Item	Item erwähnt auf Seite	Formulierung oder Bemerkung
	**Ergebnisse**	34	Präsentation der Ergebnisse für jede Delphi-Runde und der Endergebnisse	Seite 10/11	Erste Runde: Tabelle 2, siehe Ref [16]<br>Zweite Runde: Tabelle 3
V Diskussion	**Qualität der Ergebnisse**	35	Hervorhebung der Erkenntnisse des Delphi-Verfahrens	Seite 10-12, Pkt 3.2 & 3.3 Seite 13/14, Pkt. 4.1 & 4.2	„Die Delphi-Studie lieferte wichtige Hinweise dazu, wie die ALZ in der Weiterbildung gewichtet werden.“<br>„Durch die Umfrageergebnisse fließt die Meinung einer breiten Ärzteschaft in die Konzeptualisierung durch das Forscherteam ein. Die fünf finalen Kompetenzbereiche bieten deshalb eine bedürfnisorientierte Grundlage für das weitere Vorgehen…“
		36	Gültigkeit der Ergebnisse (z. B. Übertragbarkeit der Erkenntnisse)	Seite 15, Pkt. 4.3 Seite 16, 4.5	„Das CanMEDS Modell in der arbeitsplatzbasierten, klinischen Lernumgebung“<br>„Die Befragung der beiden Berufsgruppen – Ärzt*innen in Weiterbildung und Leiter*innen der WBS – macht das Ergebnis besonders wertvoll, da es sowohl die Sicht der Lernenden als auch jene der Lehrenden berücksichtigt.“
		37	Reliabilität der Ergebnisse (z. B. Split Half, Interrater-Reliabilität)	-	Trifft nicht zu
		38	Reflexion zu möglichen Limitationen (z. B. Anzahl der Expert*innen, Antwortverzerrung)	Seite 16, Pkt. 4.5	„4.5 Stärken und Grenzen der Studie“

[1] “Expert*innen” sind die Befragten des Delphi-Verfahrens. Dabei kann es sich um Personen aus der Wissenschaft, der Praxis oder um Vertreter*innen aus der Lebenswelt (z. B. Patient*innen, Familienangehörige) handeln.

[2] Der Begriff „Fragebogen“ steht für das Erhebungsinstrument, unabhängig davon, ob und wie quantitative oder qualitative Items integriert oder gewichtet sind.
