# Supplementary material for: Cross-disciplinary “General Learning Objectives” for postgraduate medical training: Prioritisation of five competency domains using a Delphi process
Source: GMS J Med Educ. 2026 Sep 15;43(6):Doc81. doi: 10.3205/zma001875 (PMC13617337; doi:10.3205/zma001875)
Supplement: Supplementary file 4 [file JME-43-81-s-004.pdf]

Attachment 4: Standardised questionnaire: General Learning Objectives – 1st round Delphi survey of postgraduate trainees and heads of postgraduate training centres

A) Personal information

1. What is your current professional situation?
- o Postgraduate trainee
- o Head of a postgraduate training centre

2. Gender:
- o Female
- o Male
- o Diverse

3. Which age group do you belong to?
- o 25 – 30
- o 31 – 40
- o 41 – 50
- o 51 – 60
- o > 60

4. Where do you work?
- o Private practice
- o District/regional hospital
- o Small cantonal hospital
- o Large cantonal hospital
- o University hospital
- o Other (please specify):

5. What specialist title do you hold or are you aiming to obtain? If you hold several specialist titles, please tick the one you consider the most important.
(Selection menu listing all specialist titles, see https://www.siwf.ch/weiterbildung/specialist title-and-schwerpunkte.cfm)

B) Assessment of the current situation

6. **Do you believe that too little emphasis is placed on General Learning Objectives (GLOs) in postgraduate medical training?** (please tick)
- o Yes
- o No
- o Do not know

Attachment 4 to Bayha E, Armstad H, Spinas G, Brodmann Maeder M, Hennel EK. *Cross-disciplinary "General Learning Objectives" for postgraduate medical training: Prioritisation fo five competency domains using a Delphi prozess*. GMS J Med Educ. 2026;43(6):Doc81. DOI: 10.3205/zma001875

7. **What do you believe are the reasons for the underemphasis on General Learning Objectives (GLOs)?** (please tick; multiple answers possible)
   - o The GLOs are unnecessary.
   - o The GLOs are not known.
   - o The GLOs lack specificity.
   - o There are too many GLOs.
   - o These are not the right GLOs.
   - o Due to the high workload, residents have too little time to implement GLOs.
   - o It is unclear how to achieve the GLOs.
   - o It is unclear how achievement of a GLO should be assessed.
   - o Other (please specify):
   - o None of the above

8. **Do you believe the current GLOs are the right ones?**
   - o Yes
   - o No
   - o Do not know

C) Evaluation of the 22 competencies required by the Swiss specialist training regulations.

The questions of this category were rated on a four-point Likert scale:
Very relevant, somewhat relevant, relevant, not relevant, do not know

Patient care:
Is able to provide patients with comprehensive advice, support and care, whilst duly respecting their autonomy.

9. How relevant is this competency for **the large majority** of physicians?

10. How relevant is this competency for physicians **in your speciality**?

Trust-based relationship:
Can build a trust-based relationship with patients and their families.

11. How relevant is this competency for **the large majority** of physicians?

12. How relevant is this competency for physicians **in your speciality**?

Communication skills:
Can provide expert information on upcoming diagnostic and therapeutic measures (informed consent discussion) and can deliver bad news in a sensitive and objective manner.

13. How relevant is this competency for **the large majority** of physicians?

14. How relevant is this competency for physicians **in your speciality**?

Diagnostics and therapy:
Can take a concise medical history, carry out a proper clinical examination, use the results to derive a working hypothesis and draw up a plan for the next steps (where possible, in accordance with the principles of evidence-based medicine).

15. How relevant is this competency for **the large majority** of physicians?

16. How relevant is this competency for physicians **in your speciality**?

Ethics:
Can apply tools for ethical decision-making and independently handle ethical issues that arise in professional routine.

17. How relevant is this competency for **the large majority** of physicians?

18. How relevant is this competency for physicians **in your speciality**?

Health economics:
Considers a reasonable cost-benefit analysis (principle of "effectiveness, appropriateness and cost-effectiveness") when rendering diagnosis, therapy and prophylaxis.

19. How relevant is this competency for **the large majority** of physicians?

20. How relevant is this competency for physicians **in your speciality**?

Patient safety/error culture:
Identifies risks, errors and complications at an early stage and manages them appropriately.

21. How relevant is this competency for **the large majority** of physicians?

22. How relevant is this competency for physicians **in your speciality**?

Recognizing one's own limits:
Consults other professionals in cases of uncertainty, if the issues lie outside the personal area of expertise, or if the patient requests it.

23. How relevant is this competency for **the large majority** of physicians?

24. How relevant is this competency for physicians **in your speciality**?

Attachment 4 to Bayha E, Armstad H, Spinas G, Brodmann Maeder M, Hennel EK. *Cross-disciplinary "General Learning Objectives" for postgraduate medical training: Prioritisation fo five competency domains using a Delphi prozess*. GMS J Med Educ. 2026;43(6):Doc81. DOI: 10.3205/zma001875

Pharmacotherapy:
Knows the indications, pharmacokinetics and relevant adverse effects and interactions of the medicines used.

25. How relevant is this competency for **the large majority** of physicians?

26. How relevant is this competency for physicians **in your speciality**?

Prescribing:
Knows the principles of prescribing and follows the safety guidelines when writing prescriptions.

27. How relevant is this competency for **the large majority** of physicians?

28. How relevant is this competency for physicians **in your speciality**?

Documentation:
Keeps ongoing records on medical history, findings, clinical course and conclusions drawn.

29. How relevant is this competency for **the large majority** of physicians?

30. How relevant is this competency for physicians **in your speciality**?

Medical information:
Maintains confidentiality of medical information.

31. How relevant is this competency for **the large majority** of physicians?

32. How relevant is this competency for physicians **in your speciality**?

Information sharing among professional colleagues:
Summarises relevant information concisely when reporting about a patient.

33. How relevant is this competency for **the large majority** of physicians?

34. How relevant is this competency for physicians **in your speciality**?

Collaboration with other healthcare professionals:
Respects the different roles and responsibilities when collaborating with other healthcare professionals.

35. How relevant is this competency for **the large majority** of physicians?

36. How relevant is this competency for physicians **in your speciality**?

Conduct towards superiors:
Recognises authority and strives to maintain an open, harmonious relationship.

37. How relevant is this competency for **the large majority** of physicians?

38. How relevant is this competency for physicians **in your speciality**?

Conduct towards staff members:
Motivates and manages subordinate staff respectfully.

39. How relevant is this competency for **the large majority** of physicians?

40. How relevant is this competency for physicians **in your speciality**?

Swiss healthcare system:
Knows its structure and organisation.

41. How relevant is this competency for **the large majority** of physicians?

42. How relevant is this competency for physicians **in your speciality**?

Swiss insurance system:
Knows and complies with the requirements of the Swiss insurance system.

43. How relevant is this competency for **the large majority** of physicians?

44. How relevant is this competency for physicians **in your speciality**?

Prevention:
Identifies health problems early and takes appropriate diagnostic and/or preventive measures for individuals and communities.

45. How relevant is this competency for **the large majority** of physicians?

46. How relevant is this competency for physicians **in your speciality**?

Postgraduate medical training and continuous professional education:
Seeks, organises and evaluates medical knowledge from relevant sources.

47. How relevant is this competency for **the large majority** of physicians?

48. How relevant is this competency for physicians **in your speciality**?

Medical research involving human subjects:
Knows the guidelines of the Swiss Academy of Medical Sciences (SAMW), the provisions of the Federal Constitution of the Swiss Confederation Art. 118b and the provisions of the Swiss Therapeutic Products Act (HMG), Swiss Federal Human Research Act (HRA) and their ordinances, (Ordinance on Clinical Trials [ClinO], Human Research Ordinance [HRO], HRA Organisation Ordinance [OrgO-HRA]) and applies them correctly.

49. How relevant is this competency for **the large majority** of physicians?

50. How relevant is this competency for physicians **in your speciality**?

Self-organisation/structuredness:
Demonstrates appropriate time and task management, sets priorities correctly.

51. How relevant is this competency for **the large majority** of physicians?

52. How relevant is this competency for physicians **in your speciality**?

D) Assessment of further topics

The questions of this category were rated on a four-point Likert scale:
Very relevant, somewhat relevant, relevant, not relevant, do not know

In recent years, additional topics have been identified that should be taught in postgraduate medical training. Please state below how relevant you deem each topic a) for the large majority of physicians and b) for physicians in your medical speciality. You may also list other topics you consider relevant a) for the large majority of physicians and b) for physicians in your speciality.

Planetary health (including climate change, sustainability, One Health)

53. How relevant is this competency for **the large majority** of physicians?

54. How relevant is this competency for physicians **in your speciality**?

Gender medicine

55. How relevant is this competency for **the large majority** of physicians?

56. How relevant is this competency for physicians **in your speciality**?

Digitalisation (including digital health, artificial intelligence)

57. How relevant is this competency for **the large majority** of physicians?

58. How relevant is this competency for physicians **in your speciality**?

Physicians health

59. How relevant is this competency for **the large majority** of physicians?

60. How relevant is this competency for physicians **in your speciality**?

Identifying and responding to domestic violence

61. How relevant is this competency for **the large majority** of physicians?

62. How relevant is this competency for physicians **in your speciality**?

New topic 1:

____

63. How relevant is this competency for **the large majority** of physicians?

64. How relevant is this competency for physicians **in your speciality**?

New topic 2:

____

65. How relevant is this competency for **the large majority** of physicians?

66. How relevant is this competency for physicians **in your speciality**?

New topic 3:

---

67. How relevant is this competency for **the large majority** of physicians?

68. How relevant is this competency for physicians **in your speciality**?

You have reached the end of the survey.

Many thanks for your valuable cooperation!

If you have any comments, please add them here:

Attachment 4 to Bayha E, Armstad H, Spinas G, Brodmann Maeder M, Hennel EK. *Cross-disciplinary "General Learning Objectives" for postgraduate medical training: Prioritisation fo five competency domains using a Delphi prozess*. GMS J Med Educ. 2026;43(6):Doc81. DOI: 10.3205/zma001875
