# Supplementary material for: Cross-disciplinary “General Learning Objectives” for postgraduate medical training: Prioritisation of five competency domains using a Delphi process
Source: GMS J Med Educ. 2026 Sep 15;43(6):Doc81. doi: 10.3205/zma001875 (PMC13617337; doi:10.3205/zma001875)
Supplement: Supplementary file 5 [file JME-43-81-s-005.pdf]

Attachment 5: Standardised questionnaire: General Learning Objectives – 2nd round Delphi survey of postgraduate trainees and heads of postgraduate training centres

A) Personal information

1. **What is your current professional situation?**
   - o Postgraduate trainee
   - o Head of a postgraduate training centre
   - o Other (please specify)

2. **Gender:**
   - o Female
   - o Male
   - o Diverse

3. **Which age group do you belong to?**
   - o 25 – 30
   - o 31 – 40
   - o 41 – 50
   - o 51 – 60
   - o > 60

4. **Where do you work?**
   - o Private practice
   - o District/regional hospital
   - o Small cantonal hospital
   - o Large cantonal hospital
   - o University hospital
   - o Other (please specify):

5. **What specialist title do you hold or are you aiming to obtain?** If you hold several specialist titles, please tick the one you consider the most important.
   Selection menu listing all specialist titles, see
   https://www.siwf.ch/weiterbildung/specialist title-and-schwerpunkte.cfm

6. **Have you already taken part in the first round of the Delphi survey?**
   - o Yes
   - o No

Attachment 5 to Bayha E, Armstad H, Spinas G, Brodmann Maeder M, Hennel EK. *Cross-disciplinary "General Learning Objectives" for postgraduate medical training: Prioritisation fo five competency domains using a Delphi prozess*. GMS J Med Educ. 2026;43(6):Doc81. DOI: 10.3205/zma001875

B) Prioritisation of General Learning Objectives

Below you will find those 12 (of 22) "regulation-compliant competencies" that were rated as "very relevant" by > 60% of the respondents or by one of the subgroups (language; gender; professional situation) in the 1st round of the Delphi survey and those 4 competencies that were most frequently cited in answers to the question about "new topics".

Patient care: Is able to provide patients with comprehensive advice, support and care, whilst duly respecting their autonomy.

Trust-based relationship: Can build a trust-based relationship with patients and their families.
Communication skills: Can provide expert information on upcoming diagnostic and therapeutic measures (informed consent discussion) and can deliver bad news in a sensitive and objective manner.

Diagnostics and therapy: Can take a concise medical history, carry out a proper clinical examination, use the results to derive a working hypothesis and draw up a plan for the next steps (where possible, in accordance with the principles of evidence-based medicine).

Patient safety/error culture: Identifies risks, errors and complications at an early stage and manages them appropriately.

Recognizing one's own limits: Consults other professionals in cases of uncertainty, if the issues lie outside the personal area of expertise, or if the patient requests it.

Pharmacotherapy: Knows the indications, pharmacokinetics and relevant adverse effects and interactions of the medicines used.

Medical information: Maintains confidentiality of medical information.

Information sharing among professional colleagues: Summarises relevant information concisely when reporting about a patient.

Collaboration with other healthcare professionals: Respects the different roles and responsibilities when collaborating with other healthcare professionals.

Conduct towards staff members: Motivates and manages subordinate staff respectfully.

Self-organisation/structuredness: Demonstrates appropriate time and task management, sets priorities correctly.

New topics:

Work-life balance: Is able to strike a balance between professional demands and personal life (family and leisure), thereby maintaining one's own health and avoiding burnout; is familiar with employment law requirements and ensures compliance.

Smarter Medicine: Recognises that, in medicine, "Less is sometimes more", and avoids treatments and diagnostic workups that offer no added benefit to patients.

Migration/transcultural competency: Can grasp and understand how individual "lifeworlds" may play out under special circumstances and in different contexts, and, from that, derive and adapt the appropriate courses of action.

Attachment 5 to Bayha E, Armstad H, Spinas G, Brodmann Maeder M, Hennel EK. *Cross-disciplinary "General Learning Objectives" for postgraduate medical training: Prioritisation fo five competency domains using a Delphi prozess*. GMS J Med Educ. 2026;43(6):Doc81. DOI: 10.3205/zma001875

Teaching/leadership: Has a solid understanding of didactics and team leadership with a view to future postgraduate medical education.

7. **Please select 8 competencies from the 16 listed above that you believe should definitely be taught as “General Learning Objectives” and rank them into priorities.**

	1st priority	2nd priority	3rd priority	4th priority	5th priority	6th priority	7th priority	8th priority
Patient care	○	○	○	○	○	○	○	○
Trust-based relationship	○	○	○	○	○	○	○	○
Communication skills	○	○	○	○	○	○	○	○
Diagnostics and therapy	○	○	○	○	○	○	○	○
Patient safety/error culture	○	○	○	○	○	○	○	○
Recognizing one’s own limits	○	○	○	○	○	○	○	○
Pharmacotherapy	○	○	○	○	○	○	○	○
Medical information	○	○	○	○	○	○	○	○
Information sharing among professional colleagues	○	○	○	○	○	○	○	○
Collaboration with other healthcare professionals	○	○	○	○	○	○	○	○
Conduct towards staff members	○	○	○	○	○	○	○	○
Self-organisation/structuredness	○	○	○	○	○	○	○	○
Work-life balance	○	○	○	○	○	○	○	○
Smarter Medicine	○	○	○	○	○	○	○	○
Migration/transcultural competency	○	○	○	○	○	○	○	○
Teaching/leadership	○	○	○	○	○	○	○	○

You have reached the end of the survey.

Many thanks for your valuable cooperation!

If you have any comments, please add them here:

Attachment 5 to Bayha E, Armstad H, Spinas G, Brodmann Maeder M, Hennel EK. *Cross-disciplinary "General Learning Objectives" for postgraduate medical training: Prioritisation fo five competency domains using a Delphi prozess*. GMS J Med Educ. 2026;43(6):Doc81. DOI: 10.3205/zma001875
